# Tracking the evolution of biomarker efficacy in SARS-CoV-2: a global meta-analyses series

**DOI:** 10.1186/s12931-026-03656-9

**Published:** 2026-04-28

**Authors:** ME Rahman Shuvo, Max Schwiening, Nikos Avramidis, Felipe Soares, Oliver Feng, Susana Abreu, Niki Veale, William Thomas, A. A. Roger Thompson, Richard J. Samworth, Nicholas W. Morrell, Malcolm G. Semple, Kenneth Baillie, Stefan J. Marciniak, Elaine Soon

**Affiliations:** 1 Department of Respiratory Medicine, Box 40, Cambridge University Hospitals NHS, Foundation Trust, Hills Road, Cambridge, CB2 0QQ UK; 2 Cambridge Institute for Medical Research, University of Cambridge, Keith Peters Building, Hills Rd, Cambridge, CB2 0XY UK; 3 Baillie Gifford Pandemic Science Hub, Centre for Inflammation Research, University of Edinburgh, 47 Little France Crescent, Edinburgh, EH16 4TJ UK; 4 Roslin Institute, University of Edinburgh, Easter Bush, Midlothian, EH25 9RG UK; 5 Department of Clinical Medicine, University of Sheffield, Beech Hill Road, Sheffield, S10 2RX UK; 6 Universidade Federal Do Rio Grande Do Sul, Farroupilha, Porto Alegre, RS 90010-150 Brazil; 7 Statistical Laboratory, Centre for Mathematical Sciences, Wilberforce Road, Cambridge, CB3 0WB UK; 8 NIHR Health Protection Research Unit in Emerging and Zoonotic Infections, Institute of Infection, Veterinary, and Ecological Sciences, University of Liverpool, Ronald Ross Building, 8 West Derby St, Liverpool, L69 7BE UK; 9 Liverpool Institute for Child Health and Wellbeing, Alder Hey Children’s Hospital, Eaton Road, Liverpool, L12 2AP UK

**Keywords:** SARS-CoV-2, Covid19, Biomarker, Mortality

## Abstract

**Background:**

Sophisticated prognostic scores have been proposed for SARS-CoV-2 but do not always perform consistently. We performed this systematic review to discover why and to investigate the impact of vaccination and viral variants.

**Methods:**

We searched the PubMed database for the keywords ‘SARS-CoV-2’ or ‘Covid19’ with ‘biomarker’ and ‘mortality’ for the baseline tranche (01/12/2019–30/06/2021) and either ‘SARS-CoV-2’ or ‘Covid19’ with ‘biomarker’ and either ‘vaccination’ or ‘variant’ from 01/12/2020 to 31/10/2023. To aggregate the data, the *meta* library in R was used, and a random effects model fitted to obtain pooled AUCs and 95% confidence intervals.

**Results:**

We screened 4,688 potential source manuscripts and included 144 in the final analyses. Biomarker effectiveness varies significantly by geographical region. Admission CRP levels were a good prognostic marker for mortality due to wild-type virus in Asian countries, with a pooled area under curve (AUC) of 0.83 (95%CI 0.80–0.85), but only an average predictor of mortality in Europe/Northern America, with a pooled AUC of 0.67 (95%CI 0.63–0.71, *P* < 0.0001). The same pattern applies to D-dimer and IL-6. Notably, urea and troponin had pooled AUCs ≥ 0.78 regardless of location, implying that end-organ damage at presentation was a key prognostic factor in wild-type SARS-CoV-2 infection. CRP, D-dimer, and IL-6 have generally declined in effectiveness in the vaccinated and variant cohorts. We note a significant lag from the pandemic advent to data availability and that the type of data varied considerably between studies.

**Conclusion:**

Biomarkers and prognostic scores should be tailored to populations. It is imperative that the infrastructure for collecting clinical data should be put in place ahead of a future pandemic so that data harvesting, collation and analysis can occur in a robust and timely manner to aid clinical decision making.

**Trial registration:**

This study was registered with PROSPERO (CRD42022366893).

**Supplementary Information:**

The online version contains supplementary material available at 10.1186/s12931-026-03656-9.

## Background

Severe acute respiratory syndrome coronavirus 2 (SARS-CoV-2) is a novel beta coronavirus of zoonotic origin first identified in Wuhan, China at the end of 2019, which led to a Public Health Emergency of International Concern between February 2020 and May 2023. SARS-CoV-2 differs from previous viral threats in showing marked transmissibility during the asymptomatic/very early symptomatic stage [1] and person-to-person transmission by both airborne and fomite routes [2]. At the beginning of the pandemic, there was no previous immunity, no known effective treatment, and no vaccine, resulting in a global death toll of >6.9 million (https://covid19.who.int/).

As a large amount of clinical data was amassed quickly (in epidemiological timeframes) in a completely naïve global population, we can track the efficacy of biomarkers and assess the impact of viral mutations and mass vaccination programs. Some prognostic scores do not do well when applied away from their derivation region/population. For example, El-Solh [3] tested 4 peer-reviewed prognostic models constructed to predict in-hospital mortality for SARS-CoV-2 patients; proposed by Chen [4]*,* Shang [5]*,* Yu [6]*,* and Wang [7]*.* Each model examined had a validation area under curve (AUC) significantly worse than that generated for their derivation cohorts. For example, the AUC of the validation cohort using the model proposed by Chen [4] was at best 0.69 (95% confidence interval [CI] 0.66–0.72) compared to the derivation AUC, which was 0.91 (95% CI 0.85–0.97). A similar pattern was noted in the other three models.

Gupta [8] tested 20 candidate prognostic models using data derived from 411 consecutively admitted adults with a PCR-confirmed diagnosis of SARS-CoV-2 in a major London hospital. Five of these models were pre-existing point-based scores not specific for Covid19 (MEWS, REMS, qSOFA, CURB65 and NEWS2) and the remainder of which were a combination of point-based scores and logistic regression models specifically derived from SARS-CoV-2 patients. None of these methods overlapped with those previously tested by El-Solh and nine of the 15 Covid-specific models had been developed in China. The most discriminating univariable predictor for in-hospital mortality was age (AUC 0.76 [95% CI 0.71–0.81]) and for in-hospital deterioration was oxygen saturation on room air (AUC 0.76 [95% CI 0.71–0.81]). More importantly, none of the models tested performed consistently better than these univariable predictors.

Interestingly, the ISARIC 4C Mortality score has performed remarkably consistently with an initial AUC of 0.78 (0.78–0.79) [9] and follow-up scores of 0.71–0.82 [10–13]. We conducted the following systematic review and meta-analyses series to see if we could explain these discrepancies, and to examine the impact of successive rounds of viral mutations and vaccination programs on biomarker efficacy.

## Methods

Our primary intention with these meta-analyses was to examine which biomarkers would be most useful in predicting mortality for the adult patient who presented to the emergency department and was then admitted under the general medical team from the community. This was done so that the biomarkers would have the widest applicability. To do this, we searched the PubMed database for the keywords ‘SARS-CoV-2’ or 'Covid19' in combination with ‘biomarker name’ and ‘mortality’. The period for the first data tranche was set from 01st December 2019 to 30th June 2021. The second search covered 01st December 2019 to 31 st October 2023 and used the following keywords:

- ‘SARS-CoV-2’ with ‘vaccination’ and ‘biomarker name’
- ‘SARS-CoV-2’ with ‘variant’ and ‘biomarker name’
- ‘Covid19’ with ‘vaccination’ and ‘biomarker name’
- ‘Covid19’ with ‘variant’ and ‘biomarker name’

We examined all papers which fulfilled the following criteria:

- Age of participants ≥ 18 years, with confirmed positivity for SARS-CoV-2,
- Availability of biomarker levels within 48 h of presentation to the emergency department from the community,
- Availability of outcome data (survival versus death), and
- Being written in English or having an English translation available.

If one of these datasets was missing the corresponding author on the study was contacted and the missing information requested. Ethical approval was obtained from the UK Integrated Research Application System (reference 281880) for analysis of the Cambridge (UK) data, in accordance with the Declaration of Helsinki. All other data has either been published and/or is held in the public domain as an anonymized resource.

We excluded reports of patients who were limited to those already admitted to intensive care. We would emphasize that we did not exclude studies where patients who were admitted generally and then sent to intensive care. We also included studies with critically ill patients who were not admitted to intensive care, whether for reasons of ITU availability or otherwise. We excluded cohorts that focused exclusively on a single disease process *e.g.* studies done in cancer or transplant patients. To see if we could include pregnant women and paediatric cases, the search algorithm was re-interrogated and all manuscripts regarding pregnant women and children extracted. 24 papers met this criterion and were examined (summarised in Supplementary Table 1 in the online supplement). However, none of them had sufficient information to add to the meta-analyses. Mortality (30-day or in-hospital) was used as the endpoint.

**Fig. 1:**
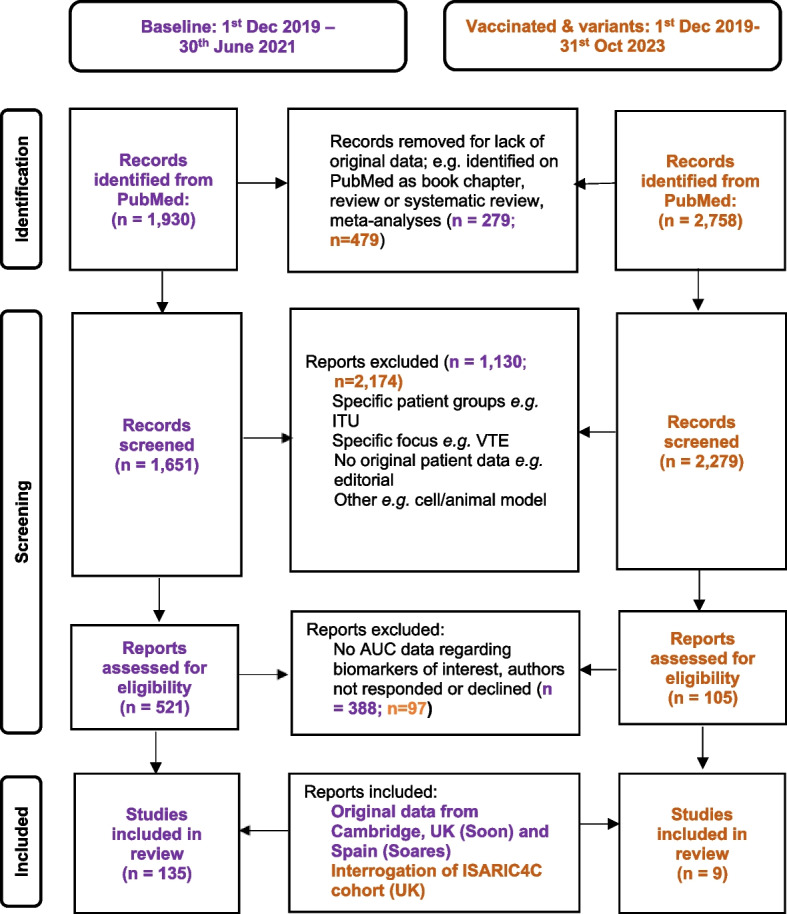
A modified PRISMA flow diagram for paper review, selection, and inclusion in these meta-analyses for both the original/wild-type virus (in purple) and for variant and vaccination-related data (in orange)

Our first survey revealed that the majority of studies were based in Asia, Europe or Northern America. We defined Asian countries as being bounded by the Indian Ocean, the Pacific Ocean, the Arctic Ocean, the Ural Mountains and the Caspian Sea and separated from the Middle East along Iran and the Caucasus Mountains. European countries were defined as being bounded by the Arctic Ocean, Atlantic Ocean, Mediterranean Sea and the Ural Mountains and the Caspian Sea. Northern American countries included Canada, the United States, Greenland and Bermuda. There were insufficient numbers of studies originating from other locations to perform adequate meta-analyses and therefore we have concentrated on two main groups: Asian countries and European/Northern American countries. This study was registered with PROSPERO (CRD42022366893); and the PRISMA flow diagram is shown in Fig. 1. The root source articles and further information are available in the online Supplement (Supplementary Figs. 1–5 and references) and at https://covid19.cimr.cam.ac.uk/.

The area under curve (AUC) was chosen as the test parameter as most of the studies analyzed had different methods of measuring their biomarkers, used different units for measurement and reported their results in different ways (e.g. by reporting odds ratios or hazard ratios per unit increase in biomarker). Therefore we pragmatically adopted the AUC as a way of estimating the efficacy of a particular biomarker for a cohort which was easy to generate, independent of the method of measurement, and which could be pooled statistically for comparison. If the AUC was not reported, we contacted the corresponding authors to request this.

### Statistical analysis

Meta-analysis of diagnostic discrimination is ideally performed using threshold-specific sensitivity and specificity, which enables hierarchical ROC modelling. However, the primary studies reported only AUC estimates and confidence intervals, precluding such approaches. We were unable to get more data as there were limits on the time and effort we could request of the source manuscript authors due to the ongoing pandemic. In this setting, transformation-based inverse-variance meta-analysis of AUCs was the only feasible quantitative synthesis.

To aggregate the data on age and biomarkers from individual studies, the *meta* library in R was used to report overall mean values and 95% confidence intervals and statistical significance of differences between mean values in the joint European and North American cohort and the Asian cohort. This analysis was based on estimates of standard errors for each study, obtained by assuming values for individual subjects were normally distributed in each study with a study-specific mean. In this way, measures of spread (IQR, SD and range) were converted into estimates of within-study standard deviations. Since the estimates of the study-specific means exhibited high levels of heterogeneity within both categories, a *random effects model* was fitted as opposed to a fixed effects model in the meta-analysis. Further details are available in the online Supplement.

To summarize, we analyzed logit-transformed AUCs, which respected the bounded nature of the statistic and leveraged the asymptotic normality underlying the confidence intervals reported in the original studies. Therefore, the pooled estimate should be interpreted as a descriptive summary of discrimination across heterogeneous populations rather than as an estimate of expected performance in a new setting. Due to the limitations inherent in this approach we have not performed further statistical analyses.

### Sensitivity analyses

Sensitivity analyses were performed by serially excluding each study to determine the implications of individual studies on the pooled AUC. No individual study had a significant implication for pooled AUCs for either European/North American or the Asian cohorts (Supplementary Tables 2–6). Note that when fitting a random effects meta-analysis model, the individual study means are assumed to be random, and the between-study heterogeneity (tau^2^) needs to be estimated. For the pooled AUC, we used a single estimate of tau^2^ based on the overall dataset (both European/North American and the Asian studies) due to small sample sizes, so removing a study affects this and thereby may shift the confidence intervals very slightly for the other subgroup.

### Patient and public involvement

After discussion with members of the public, we decided to display the root data underpinning the meta-analyses on a publicly available website (https://covid19.cimr.cam.ac.uk/). Study authors have also volunteered that statistical software is expensive and hence inaccessible. Therefore we have written a program in R which allows for calculation for the AUC of a biomarker which is free to download from the same website. Our intention is that everyone will be able to view the most effective biomarkers for their locale from the website. The website was assessed for accessibility and ease of understanding by Ms Natalie Doughty and Mr Chris Davies.

## Results

**Fig. 2:**
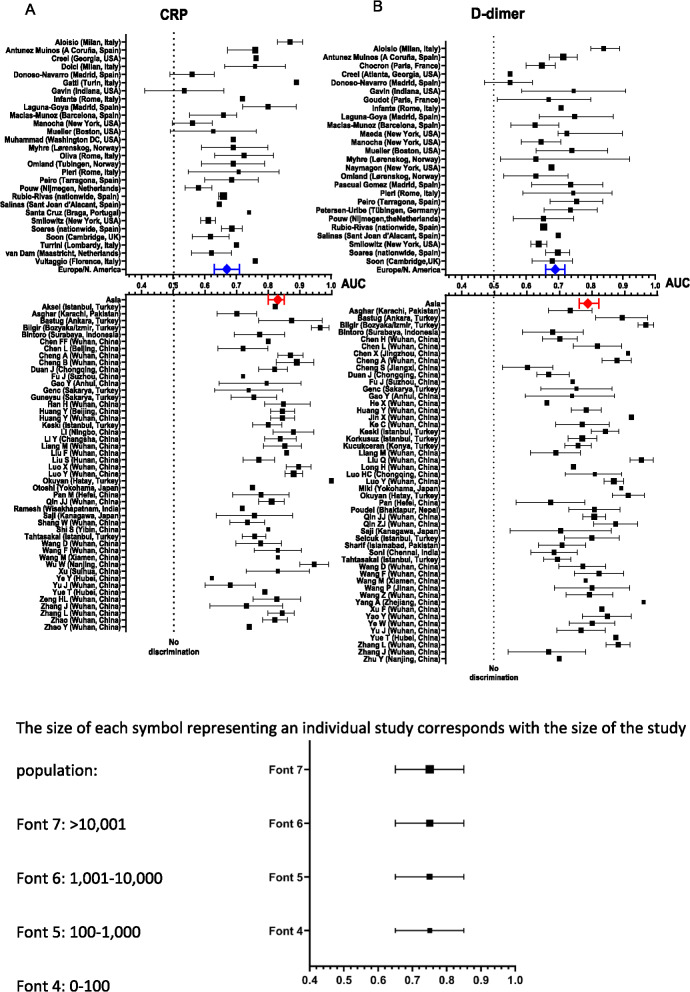
Forest plots demonstrating all individual studies contributing to the meta-analyses for (**A**) CRP and (**B**) D-dimer in Asian and European/Northern American countries for the first wild-type wave (data from Dec 2019 – June 2021). Note a significant number of studies did not quote 95% confidence intervals (18 of 75 for CRP, 14 of 78 for D-dimer, 3 of 35 for troponin, 1 of 16 for urea, 8 of 38 for IL-6) and we were unable to obtain them despite best efforts to communicate with the authors. These studies are included in Figs. 2 and 3 for completeness but are not included in calculation of the pooled AUCs. Blue diamond: pooled AUC for European/Northern American countries. Red diamond: pooled AUC for Asian countries. The size of each symbol representing an individual study corresponds with the size of the study population: Font 7: > 10,001. Font 6: 1,001–10,000. Font 5: 100–1,000. Font 4: 0–100. For the reader’s ease the data sources are arranged alphabetically within the figure

**Fig. 3:**
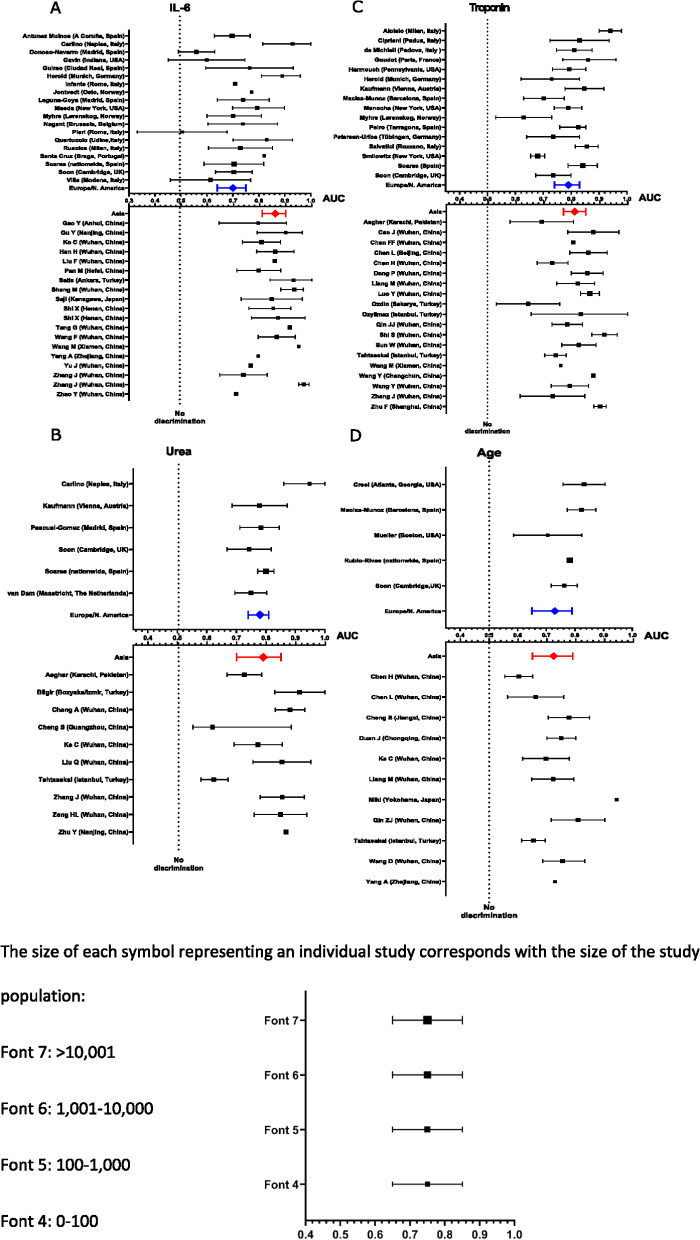
Forest plots demonstrating all individual studies contributing to the meta-analyses for (**A**) IL-6, (**B**) urea, (**C**) troponin, and (**D**) age in Asian and European and Northern American countries for the first tranche of data (from Dec 2019 – June 2021). Blue diamond: pooled AUC for European and North American countries. Red diamond: pooled AUC for Asian countries. The size of each symbol representing an individual study corresponds with the size of the study population: Font 7: > 10,001. Font 6: 1,001–10,000. Font 5: 100–1,000. Font 4: 0–100. For the reader’s ease the data sources are arranged alphabetically within the figure

We examined 1,930 articles that were published from the beginning of the SARS-CoV-2 pandemic on 01st December 2019 to 30th June 2021 for the baseline study and 2,758 papers from 01st December 2019 to 31st October 2023 to obtain vaccination and variant data. The first phase meta-analyses revealed different patterns in the effectiveness of biomarkers in different regions of the world. For example, admission CRP levels were a good prognostic marker for mortality in Asian countries, with a pooled area under curve (AUC) of 0.83 (95% confidence intervals [CI] 0.80–0.85), but only an average predictor of mortality in Europe and Northern America, with a pooled AUC of 0.67 (95% CI 0.63–0.71, *P* < 0.0001, Table 1, Fig. 2A). This was also true for admission D-dimer (Fig. 2B), with a pooled AUC of 0.78 (95% CI 0.76–0.82) in Asian countries and a pooled AUC of 0.69 (95% CI of 0.66–0.72) in European and Northern American countries and for IL-6 (Fig. 3A) levels. This may explain why some prognostic scores that are being proposed for SARS-CoV-2 do not perform evenly in different countries, as the ‘building blocks’ underpinning these prognostic scores have intrinsically different effectiveness in different populations.

**Table 1:** Results from the first meta-analyses series of wild-type virus showing pooled AUCs from the five biomarkers selected and age, with the ages and numbers of participants from each cohort

Biomarker	Location	Pooled	95% CI	*P-*value	Mean	IQR	*P-*value	No. of	No. of
		**AUC**		**for AUC**	**age**		**for age**	**patients**	**studies**
D-dimer	Asia	0.78	0.76–0.82	< 0.00001	57.9	55.8–59.9	< 0.00001	14,076	38
	Europe/	0.69	0.66–0.72		64.6	63.4–65.8		29,741	22
	N. America								
	**All**	**0.76**	**0.73–0.78**					**43,817**	**60**
CRP	Asia	0.83	0.80–0.85	< 0.00001	57.8	55.7–59.9	< 0.00001	10,407	34
	Europe/ N. America	0.67	0.63–0.71		64.8	62.8–66.7		28,693	21
	**All**	**0.78**	**0.74–0.81**					**39,100**	**55**
Urea	Asia	0.79	0.70–0.85	0.86	60.6	56.6–64.6	0.027	3,123	10
	Europe/ N. America	0.78	0.74–0.81		66.1	61.5–70.7		2,880	6
	**All**	**0.77**	**0.72–0.82**					**6,003**	**16**
Troponin	Asia	0.81	0.77–0.85	0.42	61.1	58.3–63.8	0.011	7,308	16
	Europe/ N. America	0.79	0.74–0.83		65.0	63.2–66.7		8,690	16
	**All**	**0.8**	**0.77–0.83**					**15,998**	**32**
IL-6	Asia	0.86	0.81–0.90	< 0.00001	58.2	53.6–62.9	0.033	2,993	17
	Europe/ N. America	0.70	0.64–0.75		63.5	61.1–65.9		6,362	20
	**All**	**0.78**	**0.73–0.83**					**9,355**	**37**
Age	Asia	0.73	0.65–0.79	0.0408	57.3	6.92*	0.028	2,652	9
	Europe/	0.78	0.77–0.80		62.9	2.75*		18,127	5
	N. America								
	**All**	**0.75**	**0.70–0.90**					**20,779**	**14**

*P*-values shown are for comparison between Asian and European/North American cohorts

*AUC *Area under curve, *CI *Confidence interval, *IQR *Interquartile range, *IL-6 *Interleukin-6

^*^Indicates standard deviation, entries in bold are pooled from all studies.

**Fig. 4:**
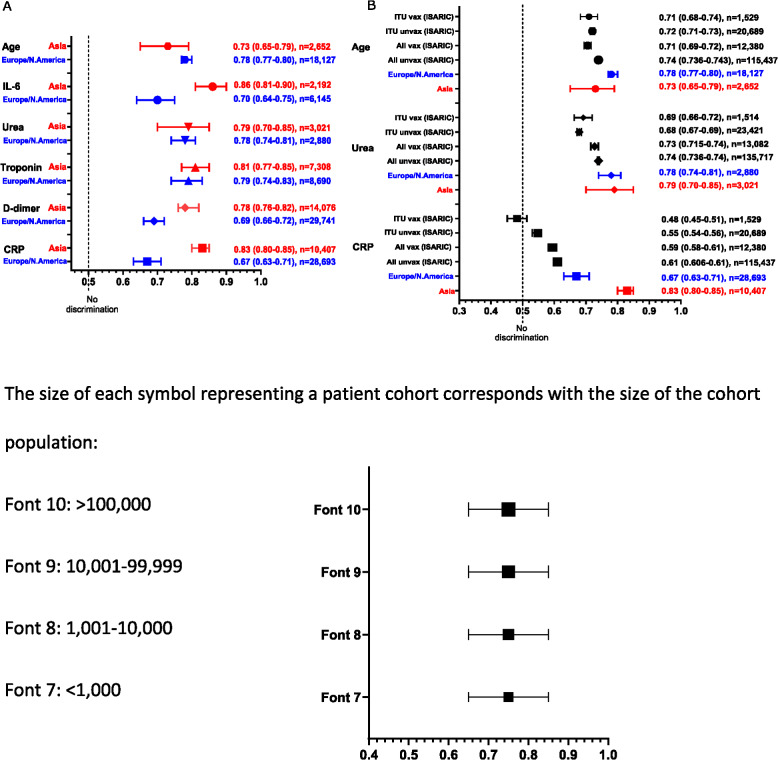
Panel (**A**) shows a summary forest plot from the meta-analyses of the first tranche of data (original/wild-type virus) with comparison of pooled area under curves for the five biomarkers being meta-analyzed (CRP, D-dimer, troponin, urea, and IL-6) and age. Panel (**B**) shows results from the ISARIC4C, GenOMICC and COG-UK consortiums (summarized in figure as ‘ISARIC’); who have data relating to intensive care admissions and vaccination status. Throughout the panels, for ease of comparison, pooled values for Asian countries are shown in red and values for European/Northern American countries are shown in blue. The size of each symbol representing a patient cohort corresponds with the size of the cohort population: Font 10: > 100,000. Font 9: 10,001–99,999. Font 8: 1,001–10,000. Font 7: < 1,000

Interestingly, urea and troponin levels had universally ‘good’ pooled AUCs (Fig. 3B-C). For example, the pooled AUC for troponin is 0.81 (95% CI 0.77–0.85) in Asia and 0.79 (95% CI 0.74–0.83) in Europe and Northern America. This implies that end-organ damage at the time of presentation was a key prognostic indicator of severity for wild-type SARS-CoV-2 infection. Age is also a reasonable prognostic biomarker with an AUC of 0.78 (95% CI 0.77–0.80) in Europe and Northern America and 0.73 (95% CI 0.65–0.79) in Asia (Fig. 3D). The results of meta-analyses for all five biomarkers and age in the first tranche (relating to wild-type virus) are summarized in Table 1 and Fig. 4A. We note that age performed better or at least as well as any of the five biochemical parameters measured (D-dimer, CRP, urea, troponin, and interleukin-6) in European and Northern American countries.

Pooling all the results from Asian, European, and Northern American studies gives a false impression of overall effectiveness for CRP, D-dimer, and IL-6 (Table 1). As an example, the pooled AUC for CRP for the entire dataset is 0.78 (95% CI 0.74–0.81). When separated into the regional blocks as previously described it becomes obvious that the Asian studies skew the results and mask the fact that admission levels of CRP, D-dimer and IL-6 were only average at predicting mortality in European and North American countries.

**Fig. 5:**
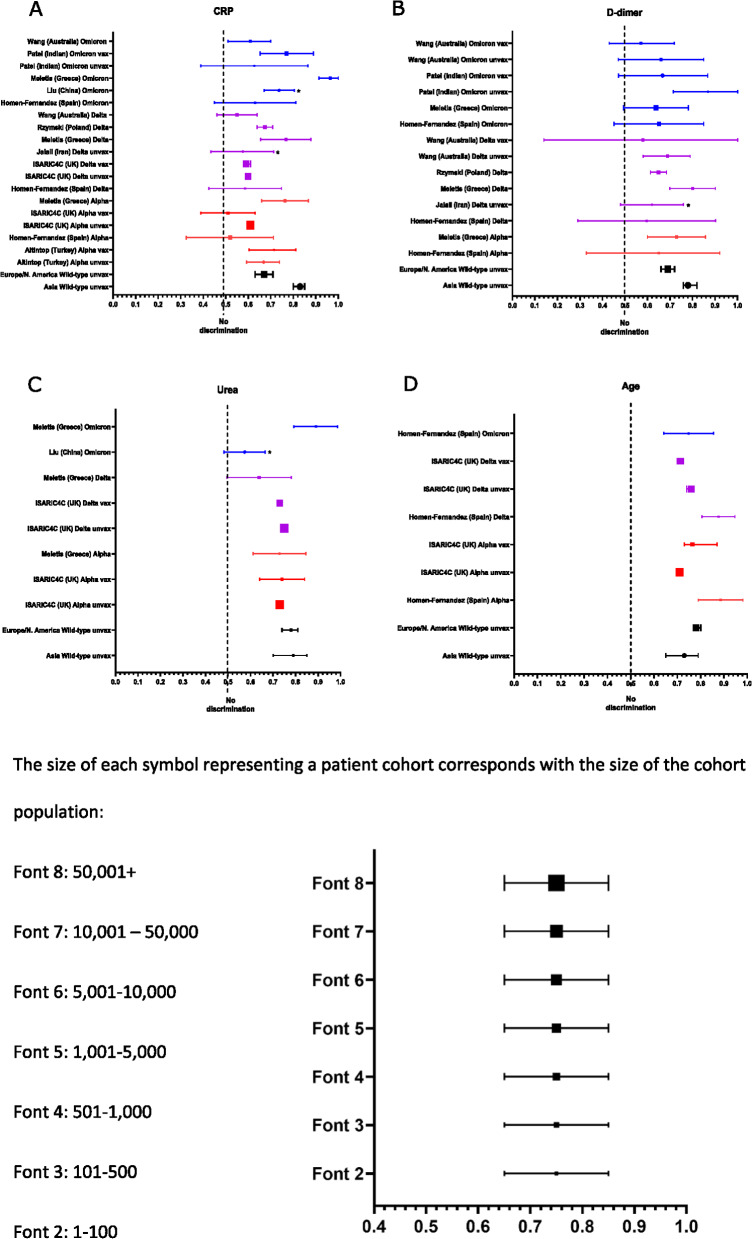
Forest plots demonstrating all individual studies with vaccination and/or variant data for (**A**) CRP, **B** D-dimer, **C** urea, and (**D**) age. Data for the Alpha variant is shown in red. Data for the Delta variant is shown in magenta. Data for the Omicron variant is shown in blue. The relevant pooled AUC from the first tranche meta-analyses (Table 1) has been included as a reference (in black). Squares indicate data from Europe/Northern America and Australia. Circles indicate data from Asian countries. *Indicate that the AUCs shown were for severe/critical illness rather than mortality. The size of each symbol representing a patient cohort corresponds with the size of the cohort population: Font 8: 50,001 + Font 7: 10,001–50,000 Font 6: 5,001–10,000 Font 5: 1,001–5,000 Font 4: 501–1,000 Font 3: 101–500 Font 2: 1–100

We expected that multiple rounds of vaccinations and ongoing mutations into different strains would significantly impact biomarker efficacy. There were insufficient numbers of studies to conduct a meta-analysis and so all the studies are collated and shown in Tables 2, and 3 and Fig. 5. The ‘inflammatory’ biomarkers (CRP, D-dimer, and IL-6) have generally declined in effectiveness in the vaccinated cohorts (Fig. 5A-B). This is particularly illustrated by our largest cohort (ISARIC4C), where the AUC for CRP in the unvaccinated Alpha cohort is 0.61 (95% CI 0.61–0.62) and in the corresponding vaccinated cohort is 0.51 (95% CI 0.39–0.63).

**Table 2:** Comparison of biomarker efficacy in vaccinated versus unvaccinated cohorts, with the relevant pooled AUC from the first tranche meta-analyses (Table 1) as a reference, with values from Asian countries in red and from European and Northern American countries in blue

Source Country	Parameter	Pooled AUC reference	2020	2021	2022
ISARIC4C [9] UK Age: 53.3 ± 14.2y	AUC CRP unvaccinated	***Wild-type*** 0.67 (0.63–0.71)	***Alpha*** 0.61 (0.61, 0.62) *n* = 68,220 Age:55.8 ± 12.9	***Delta*** 0.60 (0.59, 0.61) *n* = 47,217 Age:49.7 ± 15.2	N/A
	AUC CRP fully vaccinated	N/A	0.51	0.59	N/A
			(0.39,0.63) *n* = 489 Age:60.9 ± 9.2	(0.58, 0.61) *n* = 11,891 Age:49.7 ± 15.2	
	AUC urea unvaccinated	0.78 (0.74–0.81)	0.73	0.75	N/A
			(0.72, 0.73) *n* = 85,387 Age:56.0 ± 12.9	(0.74,0.76) *n* = 50,390 Age:49.6 ± 15.1	
	AUC urea fully vaccinated	N/A	0.74	0.73	N/A
			(0.64,0.84) *n* = 551 Age:60.5 ± 9.6	(0.72, 0.74) *n* = 12,531 Age:49.6 ± 15.0	
	AUC age unvaccinated	0.78 (0.77–0.80)	0.71 (0.71,0.72) *n* = 85,387 Age:56.0 ± 12.9	0.76 (0.76, 0.77) *n* = 50,390 Age:49.6 ± 15.1	N/A
	AUC age fully vaccinated	N/A	0.80 (0.73, 0.87) n = 551 Age:60.5 ± 9.6	0.71 (0.70, 0.72) n = 12,531 Age:49.6 ± 15.0	N/A
Wang [14] Australia Age: 64.1 ± 26.0y	AUC CRP all-cause mortality	***Wild-type*** 0.67 (0.63–0.71)*	***B.1.338*** 0.6 (0.49, 0.70) *n* = 196 Age: 69.5 (48–84)	***Delta*** 0.55 (0.46, 0.64) *n* = 419 Age: 55 (40–71)	***Omicron*** 0.61 (0.51, 0.70) *n* = 467 Age: 70 (47–82)
	AUC CRP	N/A	N/A	0.58	0.61
	unvaccinated			(0.49, 0.68)	(0.45, 0.78)
	AUC CRP	N/A	N/A	N/A	0.57
	fully vaccinated				(0.44, 0.69)
	AUC D-dimer	0.69 (0.66–0.72)*	0.69 (0.56, 0.82)	0.69 (0.59, 0.78)	0.63 (0.52, 0.73)
	all-cause mortality				
	AUC D-dimer	N/A	N/A	0.69	0.66
	unvaccinated			(0.58, 0.79)	(0.47, 0.85)
	AUC D-dimer	N/A	N/A	0.58	0.57
	fully vaccinated			(0.14, 1.00)	(0.43, 0.72)
	AUC CRP fully vaccinated	***Wild-type*** 0.67 (0.63–0.71)	N/A	***Delta*** 0.675 (0.640—0.709) *n* = 741 Age: 68.2 ± 15.8y	N/A
Rzymski [15] Poland Age: 68.2 ± 15.8y					
	AUC D-dimer fully vaccinated	0.69 (0.66–0.72)	N/A	0.649 (0.613–0.685) *n* = 741	N/A
	AUC IL-6 fully vaccinated	0.7 (0.64–0.75)	N/A	0.738 (0.695–0.778) *n* = 741	N/A
Patel [16] India Age: 54y (37–67)	AUC CRP	***Wild-type*** 0.83 (0.80–0.85)	N/A	N/A	***Omicron*** 0.627 (0.389–0.865) *n* = 79 Age: 61 (35–75)
	Unvaccinated				
	AUC CRP	N/A	N/A	N/A	0.771 (0.653–0.889) *n* = 709 Age: 53 (37–67)
	fully vaccinated				
	AUC D-dimer	0.78 (0.76–0.82)	N/A	N/A	0.868 (0.715–1.00) *n* = 79 Age: 61 (35–75)
	Unvaccinated				
	AUC D-dimer				0.667 (0.471–0.868) *n* = 709 Age: 53 (37–67)
Altintop [17] Türkiye Age: 72y (61–97)	fully vaccinated	N/A	N/A	N/A	
	AUC CRP	***Wild-type*** 0.83 (0.80–0.85)	N/A	***Original/Alpha*** 0.668 (0.592–0.739) *n* = 79	N/A
	unvaccinated				
	AUC CRP fully vaccinated	N/A	N/A	0.715 (0.603–0.811) *n* = 169	N/A

*AUC *Area under curve, *ISARIC4C* International Severe Acute Respiratory Infection Consortium Comprehensive Clinical Characterisation Collaboration

^*^Note that the European/Northern American pooled AUCs were used as the references for the Australian cohort

**Table 3:** Comparison of biomarker efficacy in viral variant cohorts, with the relevant pooled AUC from the initial meta-analyses (Table 1) as a reference, with values from Asian countries in red and from European and Northern American countries in blue

Source	Parameter	Pooled AUC	Alpha	Delta	Omicron
**Country**		**Reference**			
Homen- Fernandez [18] Spain Age: 80 ± 14y	AUC CRP	0.67 (0.63–0.71)	0.521	0.586	0.631
			0.324–0.712 *n* = 97	0.425–0.748 *n* = 98	0.450–0.811 *n* = 99
	AUC D-dimer	0.69 (0.66–0.72)	0.650	0.596	0.651
			0.328–0.921 *n* = 90	0.290–0.902 *n* = 70	0.452–0.850 *n* = 57
	AUC trop	0.81 (0.77–0.85)	0.94	0.788	0.883
			0.876–1.000 *n* = 87	0.657–0.921 *n* = 74	0.754–1.000 *n* = 34
	AUC age	0.78 (0.77–0.80)	0.886	0.877	0.749
			0.790–0.982 *n* = 100	0.806–0.947 *n* = 100	0.642–0.856 *n* = 100
Meletis [19] Greece Age: 55.2 ± 23.9y	AUC CRP	0.67 (0.63–0.71)	0.764	0.767	0.964
			0.660–0.867 *n* = 129	0.655–0.878 *n* = 130	0.914–1.000 *n* = 50
	AUC D-dimer	0.69 (0.66–0.72)	0.729	0.800	0.638
			0.600–0.858 *n* = 119	0.699–0.901 *n* = 122	0.494–0.782 *n* = 45
	AUC urea	0.77 (0.72–0.82)	0.729	0.638	0.89
			0.612–0.846 *n* = 139	0.494–0.781 *n* = 136	0.793–0.986 *n* = 34
	AUC IL-6	0.7 (0.64–0.75)	0.809	0.818	0.883
			0.708–0.911 *n* = 105	0.707–0.930 *n* = 112	0.754–1.000 *n* = 60
Azarfar [20] Iran Age: 52.4 ± 16.1y	AUC CRP*	0.83 (0.80–0.85)	N/A	0.576	N/A
				0.434–0.713 *n* = 75	
	AUC D-dimer*	0.78 (0.76–0.82)	N/A	0.620 0.481–0.760 *n* = 67	N/A
Liu [21] China Age: 50.2 ± 17.4y	AUC CRP*	0.83 (0.80–0.85)	N/A	N/A	0.737
					0.671–0.804 *n* = 311
	AUC urea*	0.79 (0.70–0.85)	N/A	N/A	0.574 0.482–0.665 *n* = 311

*AUC *Area under curve, *IL-6 *Interleukin-6

^*^Indicate that the AUCs shown were for critical illness rather than mortality

The same pattern is seen in the variant cohorts, with most studies showing that the inflammatory biomarkers have lower AUCs with variant cohorts (Table 3). Interestingly, the Azarfar cohort [20] (Delta variant), who are completely unvaccinated, have AUCs of 0.576 (95% CI 0.434–0.713) for CRP and 0.620 (95% CI 0.481–0.760) for D-dimer. This is consistent with reports that the pathogenicity of SARS-CoV-2 has decreased in the order wild-type > Delta > Omicron and that the Omicron variant in particular is associated with decreased IL-6 production [22, 23]. It is also likely that the targeting of inflammation generally (*e.g*. dexamethasone) and IL-6 pathways specifically (*e.g*. tocilizumab) has had an effect; in that we are now able to effectively neutralize at least some of the inflammatory effects of severe SARS CoV-2.

To examine this in greater detail we have interrogated data collected from the ISARIC4C [24], GenOMICC [25] and COG-UK [26] consortiums. Figure 4B shows increasing loss of efficacy for CRP from all unvaccinated in the cohort to those vaccinated to intensive care admissions. The AUCs for urea vary less between vaccinated and unvaccinated and have greater prognostic capability in every patient category examined, as does age. We think this shows that co-morbidity and/or frailty are now the leading factors in predicting susceptibility to fatal SARS-CoV-2 in the UK.

## Discussion

We demonstrate conclusively for the first time that biomarker effectiveness for mortality in SARS-CoV-2 varied significantly by geographical location. We hypothesize that biomarker efficacy in any population at this given time hinges on many factors including:

A) Population factors – the numbers vaccinated and/or previously infected, the age and co-morbidities present,
B) Variant factors such as pathogenicity and transmissibility,
C) Socio-economic factors such as access to healthcare and nutritional status,
D) Health systems factors that affect the time and severity of illness at presentation.

Prognostic biomarkers/scores are likely to perform better if individualized to specific populations. Consistent with our findings, Marino et al. [27] demonstrated that a prognostic score developed in the same country (PREDI-CO, Bartoletti et al. [28], Italy) had reasonable predictive power (AUC of 0.76, 95% CI 0.58–0.93) while a prognostic score developed in another country (Yan-XGBoost, Yan *et al.* [29]*,* China) did not perform satisfactorily (AUC of 0.57, 95% CI 0.37–0.76) when applied to their cohort.

Our observations are likely to apply to other conditions. The CURB-65 score is well-known and has been validated as a tool for predicting mortality in community-acquired pneumonia [30]. It was developed in the UK, New Zealand and the Netherlands. European studies confirm an AUC of 0.759–0.780 [31, 32]. However it performs less satisfactorily in older populations [33]. For example, Shirata et al. [34] demonstrated that CURB-65 had an AUC of 0.672 (95% CI 0.607–0.732) when applied to patients ≥ 65 years. Since Japan has one of the highest life expectancies in the world it is likely that CURB-65 would not perform as well if applied to a Japanese cohort. Interestingly, CURB-65 also performs relatively poorly when applied to Colombian patients (AUCs of 0.629–0.669 when tested against three cohorts [35]). Hincapie suggested that this may be due to the factors underpinning a significant difference in community-acquired pneumonia associated mortality (9.5% [30] *versus* 17–32% [35]).

It is not possible to know from these descriptive meta-analyses exactly why these regional differences in biomarker effectiveness existed for the first wave of SARS-CoV-2. This was likely due to a combination of multiple factors, including differences in populations (age, co-morbidities, genetics), treatment strategies, health system structures, and public health policy; which are summarized in Table 4. In the first, wild-type wave; Asian cohorts were universally younger than the European and Northern American cohorts in all five parameters we investigated (CRP, D-dimer, troponin, urea, and IL-6; Table 1). Gomez-Mesa et al. [36] showed that the point prevalence of every single co-morbidity bar diabetes (including hypertension, heart failure, chronic kidney disease, coronary artery disease and cancer) was higher in European men *versus* Asian men. It is possible that in the first wave, younger Asian patients were dying predominantly from cytokine storm (hence the marked prognostic value of the ‘inflammatory’ markers such as CRP, D-dimer, and IL-6), while in Europe and Northern America older people were dying from multi-organ failure. We note that age remained a significant prognostic factor in all ISARIC4C cohorts (all vaccinated, all unvaccinated, vaccinated in ITU, and unvaccinated in ITU; Fig. 4B) and indeed in every single cohort where reported. It is probable that age is essentially a proxy for co-morbidity and/or frailty.

**Table 4:** Summary of potential mechanistic differences between Asian and European/Northern American regions

Aspect	Asia	Europe/Northern America
Viral exposure	First exposure	‘Conditioned' by prior Asian experience
Population	Younger, fewer co-morbidities [36]	Older, more co-morbidities
Initial treatment strategy	Integrated antiviral + protocol bundles [37]	Focused on supportive care
Evidence threshold	More willingness to use observational/pragmatic evidence	Strong emphasis on large RCTs
Public health integration	Treatment integrated with containment strategy	Treatment separate from containment, emphasis on vaccination
Drug repurposing	Early broad repurposing common	Initially variable, later standardized
Guideline speed	Faster early protocol deployment	Slower adoption, frequent revisions

Other critical regional differences include different treatment strategies, health system structures, and public health policy. Asian policies were pragmatic and incorporated early use of antivirals, repurposing of other drugs, and integration with containment measures including widespread masking protocols. Zhang et al. [37] described the use of specialized ‘fever clinics’ to triage patients with respiratory symptoms using a combination of blood biomarkers (CRP, lymphocyte counts) and CT scanning to diagnose viral pneumonia which were isolated and then sent for specific SARS-CoV-2 testing. This approach was adopted due to its success in containing the severe acute respiratory syndrome (SARS) epidemic in 2003. The focus in Europe was on supportive care, mass testing including the use of home lateral flow tests [38], and episodic social contact-limiting measures (‘lockdowns’) in order to prevent local healthcare providers from being overwhelmed. In particular, use of repurposed drugs was more restricted to trial formats in Europe, with strong emphasis on large RCTs and widespread adoption of dexamethasone after publication of the RECOVERY trial [39].

Multinational practice data have also demonstrated variation in respiratory support strategies and ITU admission rates. These include higher reported use of prone positioning in parts of Asia and Europe (≈70–85%) compared with North America (≈50–70%). ITU admission rates also differed: 35% in the USA, 17% in the UK, and 14% in China [40]; and in many countries was influenced by ITU capacity due to an overwhelming need. The corresponding ITU mortality was 29% (USA), 33% (UK) and 24% in China. In parallel, pandemic-associated disruption of non-COVID care was more pronounced in Asia, where Teglia et al. [41] indicate treatment reductions in cancer services approaching − 42% (95% CI: −50 to −35%) compared with − 35% (95% CI: −47 to −22%) in North America and − 8% (95% CI: −16 to −0.2%) in Europe, suggesting greater system-level interruption and diagnostic or therapeutic delay. Mechanistically, these differences likely arise from (i) variable ICU capacity and escalation criteria, (ii) differential early reliance on imaging versus molecular testing, (iii) intensity of lockdown measures affecting care continuity, and (iv) population-level risk structures. Together, the literature supports a region-adaptive framework for clinical management and biomarker selection, recognizing that inflammatory phenotyping may be more informative in settings with younger populations and hyperinflammatory presentations, whereas end organ-damage-oriented risk stratification may be prioritized in systems with older patients and high comorbidity burden.

This study has several limitations. First, insufficient numbers of studies were located in continents other than Asia and Northern America/Europe to perform an adequate meta-analysis for the first tranche (wild-type virus). There were also inadequate numbers of published studies in vaccinated and variant cohorts for meta-analyses and so we have elected to collate and show the results of each study individually. We also do not have any data to make inferences for the pregnant or paediatric populations. Due to the nature of our representative characteristic (the area under curve for the receiver operator characteristic) we are unable to suggest threshold values for the biomarkers in question. Our pooled estimate of AUCs should be interpreted as a descriptive summary of discrimination across heterogeneous populations rather than as an estimate of expected performance in a new setting; since the robustness of our statistical approach has been limited by the data available. The biomarker detection time varies in the studies we have analysed and collated and this also introduces further uncertainty.

Finally and most importantly, we need to be better prepared to face another pandemic. There was a significant lag between data availability and the clinical need for it; and the data itself was not collected in a consistent manner which would have allowed for a more robust analysis. For example it took over a year for data collection for our initial wild-type tranche to be completed, by which time SARS-CoV-2 had evolved sufficiently for the original analyses to be outdated. Access to statistical software was also limited and this led us to write a free-to-use program in R to calculate AUC (available to download at https://covid19.cimr.cam.ac.uk/).

The simplest and most practical way to overcome these issues would be to set up information frameworks ahead of time, ideally online, in an automated manner and with ethical permissions already in place. In this way, data collection can be standardized, rather than growing organically as was the case with the SARS-CoV-2 pandemic. The availability of such standardized data would facilitate rapid collation and robust meta-analyses and would reduce the time and effort required from clinicians and researchers, which will be in short supply during a pandemic. We strongly recommend the use of open-source tools to facilitate this, such as the WHO/ISARIC Clinical Characterization Protocol (https://isaric.net/ccp/) [42, 43]. This protocol is free to use and investigators will fully control their data and samples. Specific centers could also be selected to gather information on groups such as pregnant women and children, who are too often left out of studies. This system would allow the rapid identification of population-specific biomarkers and the organization of clinical trials by pinpointing ‘at-risk’ groups who could be targeted early for vaccination or intervention programs.

## Conclusions

Biomarkers and prognostic scores should be tailored to particular populations. It is imperative that the infrastructure for collecting clinical data should be put in place ahead of a future pandemic so that data harvesting, collation and analysis can occur in a robust manner, and the results available in time to assist clinical decision making.

## Supplementary Information

Supplementary Material 1.

## Data Availability

All the root studies are fully cited in the references, supplemental data and at [https://covid19.cimr.cam.ac.uk] (). The datasets generated and analyzed during the current study are available from the corresponding author on reasonable request.

## Abbreviations

AUC: Area under curve
CI: Confidence interval
COG-UK: Covid-19 Genomics UK Consortium
CRP: C-reactive protein
IL-6: Interleukin-6
IQR: Interquartile range
ISARIC: International Severe Acute Respiratory and emerging Infection Consortium
ITU: Intensive care unit
SARS-CoV-2: Severe acute respiratory syndrome coronavirus 2
WHO: World Health Organization
