# Supplementary material for: Tracking the evolution of biomarker efficacy in SARS-CoV-2: a global meta-analyses series
Source: Respir Res. 2026 Apr 28;27:265. doi: 10.1186/s12931-026-03656-9 (PMC13317354; doi:10.1186/s12931-026-03656-9)
Supplement: Supplementary file 1 — Supplementary Material 1. [file 12931_2026_3656_MOESM1_ESM.pdf]

Online Supplement

Tracking the evolution of biomarker efficacy in SARS-CoV-2: a global meta-analyses series

ME Rahman Shuvo, Max Schwiening, Nikos Avramidis, Felipe Soares, Oliver Feng, Susana Abreu, Niki Veale, William Thomas, AA Roger Thompson, Richard J Samworth, Nicholas W Morrell, Malcolm G Semple, Kenneth Baillie, Stefan J. Marciniak, Elaine Soon.

Supplementary Statistical Methods:

Background: Elevated levels of each of these biomarkers are indicative of different acute disease processes in severely ill patients, and are therefore associated with different causes of death. For example, Covid can cause blood clots that lead to higher levels of D-dimer, while IL-6 and CRP are secreted after inflammatory events (e.g. cytokine storms). Troponin is released into the bloodstream when cardiac muscle is damaged and urea levels are monitored because Covid can affect renal function.

Raw data and pre-processing: In each study, we do not have access to individual patient data, but the following measurements are reported:

Study leader and country (West or East); *Cohort size*; *AUC and associated 95% confidence interval*; *Age*: *mean* and *spread*; *Biomarker level: mean* and *spread*; *type of outcome measured* (mortality or critical illness)

A popular approach for computing confidence intervals for AUC is by bootstrapping, but some studies may have employed alternative methods depending on the software packages they used.

Converting spread measurements or confidence intervals into a standard error for the mean estimate: The spread is given as either a SD estimate $\hat{\sigma}$, a 95% confidence interval $[\hat{l}, \hat{u}]$, an IQR $[\hat{q}, \hat{Q}]$ or a range $[\hat{m}, \hat{M}]$. Under the assumption that the age and biomarker levels are normally distributed, we converted the IQR and range measurements into SD estimates via

$$\hat{\sigma}^{\mathrm{CI}} = \frac{\sqrt{n}(\hat{u} - \hat{l})}{2\Phi^{-1}(0.95)}, \quad \hat{\sigma}^{\mathrm{IQR}} = \frac{\hat{Q} - \hat{q}}{2\Phi^{-1}(3/4)} \quad \text{and} \quad \hat{\sigma}^{\mathrm{range}} = \frac{\hat{M} - \hat{m}}{2\sqrt{2\log n}}, \tag{1}$$

where n is the cohort size. The standard error of the mean estimate was then calculated as $\hat{\sigma}/\sqrt{n}$.

Pooling data from subgroups: Sometimes, the age and biomarker information are reported separately for different subgroups of patients (e.g. in different hospitals or wards) of sizes $n_1, \ldots, n_K$ (with $\sum_{k=1}^{K} n_k = n$), in which case we aggregated the individual mean and variance estimates $\hat{\mu}_1, \ldots, \hat{\mu}_K$ and $\hat{\sigma}_1^2, \ldots, \hat{\sigma}_K^2$ via

$$\hat{\mu} = \frac{1}{n}\sum_{k=1}^{K} n_k \hat{\mu}_k \quad \text{and} \quad \hat{\sigma}^2 = \frac{1}{n}\sum_{k=1}^{K} n_k\big(\hat{\sigma}_k^2 + (\hat{\mu}_k - \hat{\mu})^2\big).$$

The reasoning behind this is that if $Z_{k1}, \ldots, Z_{kn_k}$ are random variables with mean μ_k and variance σ_k^2 for $1 \leq k \leq K$, then letting Z be drawn uniformly at random from $\{Z_{k\ell} : 1 \leq k \leq$

K, $1 \le \ell \le n_k\}$, we have

$$\mu = \mathbb{E}(Z) = \frac{1}{n}\sum_{k=1}^{k} n_k \mu_k \quad \text{and} \quad \sigma^2 = \mathbb{E}\big((Z-\mu)^2\big) = \frac{1}{n}\sum_{k=1}^{K} n_k\big(\sigma_k^2 + (\mu_k - \mu)^2\big).$$

Statistical meta-analysis: Ideally, we would build a logistic regression model that includes age, gender, levels of different biomarkers, country (West or East), type of patient (ICU or non-ICU) etc. as covariates. However, because individual patient data is not available, the current approach is to study the biomarkers individually and determine whether or not there is any significant difference in

- Ability of a single biomarker to predict mortality, as measured by AUC;
- Biomarker level at admission;
- Age

between Western and Eastern cohorts.

Biomarker level and age: Since the mean estimates for individual studies were found to exhibit high levels of heterogeneity within both the Western and Eastern parts of the overall dataset, we fitted the *random effects model*

$$\bar{Y}_{ij} = \alpha_i + b_{ij} + \varepsilon_{ij} \quad \text{for } i = 1, 2,\ j = 1, \ldots, N_i \tag{2}$$

using the `R` package `meta`. Here, N_1 and N_2 are the total number of studies from West ($i = 1$) and East ($i = 2$) respectively, α_1, α_2 are the (fixed) baseline means for West and East, $\bar{Y}_{ij}$ is the mean for the jth study in region i (with n_{ij} participants), $\{b_{ij} \sim N(0, \tau_i^2) : 1 \le i \le 2,\ 1 \le j \le N_i\}$ are independent *random effects*, and $\varepsilon_{ij} \sim N(0, \sigma_{ij}^2/n_{ij})$ are independent noise variables.

The **output** from each meta-analysis consists of estimates $\hat{\alpha}_1, \hat{\alpha}_2$ of the Western and Eastern baseline means, together with 95% confidence intervals for both, as well as a p-value for testing

$$H_0 : \alpha_1 = \alpha_2 \quad \text{against} \quad H_1 : \alpha_1 \neq \alpha_2.$$

Further statistical details: Consider first the simpler model where the σ_{ij} are known and $\tau_i = 0$ for $i = 1, 2$ (i.e. there are no random effects). Then the MLEs of α_1 and α_2 are given by

$$\tilde{\alpha}_i = \underset{\mu}{\text{argmin}} \sum_{j=1}^{N_i} \frac{n_{ij}}{2\sigma_{ij}^2}(\bar{Y}_{ij} - \mu)^2 = \frac{\sum_{j=1}^{N_i} w_{ij}\bar{Y}_{ij}}{\sum_{j=1}^{N_i} w_{ij}}$$

for $i = 1, 2$, where $w_{ij} = n_{ij}/\sigma_{ij}^2$ are the *inverse variance weights*. In the model (2), the estimates take the form

$$\hat{\alpha}_i = \frac{\sum_{j=1}^{N_i} \hat{w}_{ij}\bar{Y}_{ij}}{\sum_{j=1}^{N_i} \hat{w}_{ij}} \quad \text{with} \quad \hat{w}_{ij} = \left(\frac{\hat{\sigma}_{ij}^2}{n_{ij}} + \hat{\tau}_i^2\right)^{-1},$$

where the estimates $\hat{\tau}_1^2, \hat{\tau}_2^2$ of the variances of the random effects are obtained by e.g. MLE or restricted maximum likelihood (REML), and the variance estimates $\hat{\sigma}_{ij}^2$ from the individual studies are plugged in as if they were the population quantities.

AUC scores for single biomarkers: The AUC scores for a given biomarker take values in $[0, 1]$ and are not normally distributed, so following the approach of Debray et al. (2017, 2019), we first

logit-transformed the reported AUC scores p_{ij} and confidence intervals $[p_{ij}^l, p_{ij}^u]$ for individual studies, and then obtained a standard error for $\mathrm{logit}(p_{ij}) = \log\big(p_{ij}/(1-p_{ij})\big)$ via (1). We then fitted a random effects model (2) with $\mathrm{logit}(p_{ij})$ in place of $\bar{Y}_{ij}$, and then applied the inverse logit transform $t \mapsto 1/(1+e^{-t})$ to the resulting estimates to obtain pooled AUCs (and associated 95% confidence intervals) for the Western, Eastern and overall datasets respectively. For the biomarker under consideration, this analysis also yields a p-value for the significance of the difference between the AUC scores in Western and Eastern countries.

Biomarker	Specialist group	First author	Pubmed ID	Comment
CRP	Children	Gonzalez-Dambrauskas S	35599855	No biomarker data available in manuscript
CRP	Children	Sugunan S	33876782	N=32, no deaths
CRP and urea	Children	Yılmaz K	34107134	N=164, 2 deaths, mortality=1.22%
CRP	Children	Diaz F	34869111	N=66, mortality=4.5%; n=46 from MedRxiv
CRP	Pregnant	Di Guardo F	33389111	No AUC data, lead author contacted
D-dimer	Children	Al-Ghafry M	33098753	Case series of 8 patients, no deaths
D-dimer and CRP	Children	Dewi R	33857609	No AUC data, lead author contacted
D-dimer	Children	Farias ECF	32876282	Case report of one child
D-dimer	Children	Fernández-Sarmiento J	34259765	Review, no original patient data
D-dimer	Children	García-Salido A	32459790	Case series covering 11 patients, no deaths
D-dimer	children	Lee PY	32701511	Case series covering 28 patients, no deaths
D-dimer	Children	Ren GL	33593415	Case series covering 40 patients, no deaths
D-dimer	Children	Tolunay O	34028528	Case series covering 52 patients, no deaths
D-dimer	Children	Yuliarto S	34172701	Case report of one child
IL-6	Children	Diaz F	34869111	N=66, mortality=4.5%; n=46 from MedRxiv
IL-6	Children	Kwak JH	33445833	Review, no original patient data
IL-6	Children	Valverde I	33166189	Only 1 death in 286 children, mort rate =0.35%
IL-6	Children	Wang X	32844290	Case series describing 3 patients
Troponin	Children	Cantor A	32810894	No deaths
Troponin	Children	Sanil Y	33957250	No deaths
Troponin	Children	Valverde I	33166189	1 death in 286 patients, mort rate of 0.35%
Troponin	Children	Yuliarto S	34172701	Case report of one patient
Troponin	Children	Pereira MFB	32844958	In Brazil so out of area
Troponin	Pregnant	Mercedes BR	33098814	2 deaths in 154 patients, mort rate of 1.3%

Supplementary Table 1

Studies covering pregnant women and children from the original first-wave meta-analyses.

Studies with potential to contribute to the meta-analyses are highlighted in blue.

Pooled AUC Europe/NA	Lower Limit Europe/NA	Upper Limit Europe/NA	Pooled AUC Asia	Lower Limit Asia	Upper Limit Asia	p-value	Study omitted
**0.6728**	**0.6294**	**0.7133**	**0.8267**	**0.7958**	**0.8538**	**4.629E-10**	**None**
0.6759	0.6306	0.7181	0.8267	0.7958	0.8538	1.9685E-09	Soon (Cambridge)
0.6722	0.6262	0.7152	0.8267	0.7958	0.8538	1.1395E-09	Soares (Spain)
0.6757	0.6304	0.7180	0.8267	0.7958	0.8538	1.9182E-09	van Dam (Netherlands)
0.6566	0.6220	0.6896	0.8267	0.7958	0.8538	8.6597E-14	Aloisio (Italy)
0.6724	0.6266	0.7151	0.8267	0.7958	0.8538	1.0607E-09	Peiro (Spain)
0.6785	0.6348	0.7193	0.8267	0.7958	0.8538	1.976E-09	Manocha (USA)
0.6714	0.6257	0.7142	0.8267	0.7958	0.8538	8.5351E-10	Montrucchio (Italy)
0.6718	0.6264	0.7141	0.8267	0.7958	0.8538	7.7371E-10	Pieri (Italy)
0.6670	0.6223	0.7089	0.8267	0.7958	0.8538	1.957E-10	Muinos (Spain)
0.6779	0.6347	0.7184	0.8267	0.7958	0.8538	1.4483E-09	Kara (USA)
0.6669	0.6235	0.7076	0.8267	0.7958	0.8538	1.078E-10	Laguna-Goya (Spain)
0.6722	0.6266	0.7148	0.8267	0.7958	0.8538	9.6236E-10	Myrhe (Norway)
0.6784	0.6347	0.7192	0.8267	0.7958	0.8538	1.928E-09	Donoso-Navarro (Spain)
0.6753	0.6299	0.7176	0.8267	0.7958	0.8538	1.7903E-09	Macias-Munoz (Spain)
0.6739	0.6279	0.7168	0.8267	0.7958	0.8538	1.6296E-09	Rubio-Rivas (Spain)
0.6746	0.6294	0.7167	0.8267	0.7958	0.8538	1.3959E-09	Mueller (USA)
0.6780	0.6337	0.7193	0.8267	0.7958	0.8538	2.1939E-09	Pouw (Netherlands)
0.6766	0.6314	0.7187	0.8267	0.7958	0.8538	2.2088E-09	Smilowitz (USA)
0.6722	0.6265	0.7148	0.8267	0.7958	0.8538	9.7217E-10	Omland (Norway)
0.6686	0.6241	0.7103	0.8267	0.7958	0.8538	2.6866E-10	Dolci (Italy)
0.6705	0.6251	0.7130	0.8267	0.7958	0.8538	6.0326E-10	Oliva (Italy)
0.6728	0.6294	0.7133	0.8297	0.8001	0.8557	8.7022E-11	Asghar (Pakistan)
0.6728	0.6294	0.7133	0.8247	0.7930	0.8524	1.1669E-09	Cheng (China)
0.6728	0.6294	0.7133	0.8293	0.7980	0.8567	3.2389E-10	Tahtasakal (Turkey)
0.6728	0.6294	0.7133	0.8294	0.7989	0.8562	1.8498E-10	Chen L (China)
0.6728	0.6294	0.7133	0.8288	0.7977	0.8561	3.2087E-10	Zhang J (China)
0.6728	0.6294	0.7133	0.8264	0.7939	0.8547	1.3448E-09	Huang (China)
0.6728	0.6294	0.7133	0.8249	0.7928	0.8530	1.4766E-09	Cheng A (China)
0.6728	0.6294	0.7133	0.8237	0.7919	0.8516	1.6089E-09	Luo Y (China)
0.6728	0.6294	0.7133	0.8282	0.7959	0.8563	7.6537E-10	Keski (Turkey)
0.6728	0.6294	0.7133	0.8265	0.7940	0.8548	1.3139E-09	Zhang L (China)
0.6728	0.6294	0.7133	0.8261	0.7944	0.8539	8.8744E-10	Bastug (Turkey)
0.6728	0.6294	0.7133	0.8283	0.7965	0.8561	5.5814E-10	Pan (China)
0.6728	0.6294	0.7133	0.8231	0.7919	0.8506	1.3213E-09	Luo (China)
0.6728	0.6294	0.7133	0.8262	0.7939	0.8543	1.2356E-09	Liang (China)
0.6728	0.6294	0.7133	0.8264	0.7943	0.8545	1.0568E-09	Han (China)
0.6728	0.6294	0.7133	0.8275	0.7951	0.8558	1.0157E-09	Duan (China)
0.6728	0.6294	0.7133	0.8286	0.7968	0.8564	5.2922E-10	Wang D (China)
0.6728	0.6294	0.7133	0.8276	0.7957	0.8554	6.8295E-10	Gao Y (China)
0.6728	0.6294	0.7133	0.8234	0.7986	0.8457	1.4571E-11	Okuyan (Turkey)
0.6728	0.6294	0.7133	0.8288	0.7976	0.8561	3.3895E-10	Genc (Turkey)
0.6728	0.6294	0.7133	0.8268	0.7943	0.8550	1.1898E-09	Li Y (China)
0.6728	0.6294	0.7133	0.8228	0.7931	0.8490	5.3769E-10	Wu W (China)
0.6728	0.6294	0.7133	0.8279	0.7955	0.8561	8.9683E-10	Qin JJ (China)
0.6728	0.6294	0.7133	0.8271	0.7949	0.8552	9.6175E-10	Zeng HL (China)
0.6728	0.6294	0.7133	0.8286	0.7968	0.8563	5.158E-10	Bintoro (Indonesia)
0.6728	0.6294	0.7133	0.8268	0.7948	0.8547	9.1147E-10	Xu JB (China)
0.6728	0.6294	0.7133	0.8275	0.7950	0.8558	1.0443E-09	Zhao Y (China)
0.6728	0.6294	0.7133	0.8282	0.7965	0.8558	5.3729E-10	Saji (Japan)

0.6728	0.6294	0.7133	0.8223	0.7933	0.8481	4.0471E-10	Bilgir (Turkey)
0.6728	0.6294	0.7133	0.8290	0.7976	0.8565	3.6033E-10	Guneysu (Turkey)
0.6728	0.6294	0.7133	0.8253	0.7934	0.8531	1.1591E-09	Li J (China)
0.6728	0.6294	0.7133	0.8268	0.7945	0.8549	1.0518E-09	Wang F (China)

Supplementary Table 2

Pooled AUCs for CRP calculated with omitting a single study at a time. The first line (in bold) shows the summary from all studies with no omissions.

Pooled AUC Europe/NA	Lower Limit Europe/NA	Upper Limit Europe/NA	Pooled AUC Asia	Lower Limit Asia	Upper Limit Asia	p-value	Study omitted
**0.6884**	**0.6566**	**0.7185**	**0.7895**	**0.7564**	**0.8192**	**5.3114E-06**	**None**
0.6886	0.6557	0.7197	0.7895	0.7564	0.8192	6.9417E-06	Soon (Cambridge)
0.6882	0.6543	0.7202	0.7895	0.7564	0.8192	8.2452E-06	Soares (Spain)
0.6866	0.6536	0.7179	0.7895	0.7564	0.8192	4.8748E-06	Gomez (Spain)
0.6874	0.6543	0.7187	0.7895	0.7564	0.8192	5.75E-06	Maeda (USA)
0.6718	0.6473	0.6954	0.7895	0.7564	0.8192	1.5102E-08	Aloisio (Italy)
0.6849	0.6522	0.7159	0.7895	0.7564	0.8192	3.2088E-06	Peiro (Spain)
0.6892	0.6565	0.7202	0.7895	0.7564	0.8192	7.658E-06	Myrhe (Norway)
0.6914	0.6582	0.7228	0.7895	0.7564	0.8192	1.3108E-05	Manocha (USA)
0.6869	0.6540	0.7180	0.7895	0.7564	0.8192	4.9141E-06	Pieri (Italy)
0.6868	0.6531	0.7188	0.7895	0.7564	0.8192	6.0822E-06	Muinos (Spain)
0.6873	0.6545	0.7184	0.7895	0.7564	0.8192	5.3418E-06	Gavin (USA)
0.6864	0.6536	0.7175	0.7895	0.7564	0.8192	4.4513E-06	Laguna-Goya (Spain)
0.6915	0.6582	0.7230	0.7895	0.7564	0.8192	1.3767E-05	Chocron (France)
0.6921	0.6594	0.7230	0.7895	0.7564	0.8192	1.3386E-05	Macias-Munoz (Spain)
0.6949	0.6655	0.7228	0.7895	0.7564	0.8192	1.1215E-05	Donoso-Navarro (Spain)
0.6892	0.6561	0.7205	0.7895	0.7564	0.8192	8.3615E-06	Goudot (France)
0.6916	0.6580	0.7232	0.7895	0.7564	0.8192	1.4498E-05	Rubio-Rivas (Spain)
0.6866	0.6536	0.7178	0.7895	0.7564	0.8192	4.762E-06	Mueller (USA)
0.6905	0.6572	0.7218	0.7895	0.7564	0.8192	1.086E-05	Pouw (Netherlands)
0.6925	0.6593	0.7237	0.7895	0.7564	0.8192	1.5618E-05	Smilowitz (USA)
0.6913	0.6585	0.7223	0.7895	0.7564	0.8192	1.1715E-05	Omland (Norway)
0.6861	0.6529	0.7174	0.7895	0.7564	0.8192	4.4878E-06	Petersen-Uribe (Germany)
0.6884	0.6566	0.7185	0.7880	0.7539	0.8184	1.0166E-05	Yao (China)
0.6884	0.6566	0.7185	0.7913	0.7574	0.8216	4.9517E-06	Asghar (Pakistan)
0.6884	0.6566	0.7185	0.7918	0.7582	0.8219	4.0421E-06	Sharif (Pakistan)
0.6884	0.6566	0.7185	0.7923	0.7589	0.8222	3.4047E-06	Tahtasakal (Turkey)
0.6884	0.6566	0.7185	0.7903	0.7562	0.8208	6.5837E-06	Ke (China)
0.6884	0.6566	0.7185	0.7908	0.7567	0.8212	5.9499E-06	Kucukceran (Turkey)
0.6884	0.6566	0.7185	0.7895	0.7554	0.8200	7.8759E-06	Selcuk (Turkey)
0.6884	0.6566	0.7185	0.7923	0.7590	0.8221	3.2284E-06	Soni (India)
0.6884	0.6566	0.7185	0.7890	0.7549	0.8196	8.6246E-06	Luo HC (China)
0.6884	0.6566	0.7185	0.7890	0.7548	0.8196	8.7767E-06	Chen L (China)
0.6884	0.6566	0.7185	0.7897	0.7555	0.8203	7.659E-06	Wang Z (China)
0.6884	0.6566	0.7185	0.7921	0.7586	0.8221	3.6913E-06	Chen H (China)
0.6884	0.6566	0.7185	0.7872	0.7534	0.8175	1.086E-05	Qin ZJ (China)
0.6884	0.6566	0.7185	0.7920	0.7587	0.8218	3.4374E-06	Zhang J (China)
0.6884	0.6566	0.7185	0.7901	0.7558	0.8206	7.2557E-06	Huang Y (China)
0.6884	0.6566	0.7185	0.7861	0.7524	0.8164	1.2974E-05	Cheng A (China)
0.6884	0.6566	0.7185	0.7895	0.7553	0.8200	8.0692E-06	Ye W (China)
0.6884	0.6566	0.7185	0.7844	0.7526	0.8130	8.731E-06	Liu Q (China)
0.6884	0.6566	0.7185	0.7865	0.7525	0.8169	1.3153E-05	Luo Y (China)
0.6884	0.6566	0.7185	0.7878	0.7536	0.8184	1.1083E-05	Keski (Turkey)
0.6884	0.6566	0.7185	0.7857	0.7520	0.8159	1.3756E-05	Zhang L (China)

0.6884	0.6566	0.7185	0.7921	0.7587	0.8220	3.5069E-06	Liang (China)
0.6884	0.6566	0.7185	0.7873	0.7537	0.8173	9.66E-06	Bastug (Turkey)
0.6884	0.6566	0.7185	0.7895	0.7554	0.8199	7.5685E-06	Wang P (China)
0.6884	0.6566	0.7185	0.7936	0.7615	0.8224	1.456E-06	Cheng S (China)
0.6884	0.6566	0.7185	0.7902	0.7561	0.8207	6.8324E-06	Wang D (China)
0.6884	0.6566	0.7185	0.7905	0.7566	0.8208	5.8778E-06	Gao Y (China)
0.6884	0.6566	0.7185	0.7932	0.7606	0.8225	2.0196E-06	Duan (China)
0.6884	0.6566	0.7185	0.7922	0.7589	0.8220	3.226E-06	M Pan (China)
0.6884	0.6566	0.7185	0.7891	0.7548	0.8198	9.0186E-06	Qin JJ (China)
0.6884	0.6566	0.7185	0.7893	0.7551	0.8198	8.295E-06	Poudel (Nepal)
0.6884	0.6566	0.7185	0.7853	0.7522	0.8150	1.1949E-05	Okuyan (Turkey)
0.6884	0.6566	0.7185	0.7906	0.7566	0.8209	5.8778E-06	Genc (Turkey)
0.6884	0.6566	0.7185	0.7904	0.7563	0.8209	6.6122E-06	Korkusuz (Turkey)
0.6884	0.6566	0.7185	0.7922	0.7589	0.8220	3.2827E-06	Bintoro (Surabaya, Indonesia)
0.6884	0.6566	0.7185	0.7909	0.7572	0.8211	5.0085E-06	Saji (Kanagawa, Japan)
0.6884	0.6566	0.7185	0.7821	0.7521	0.8095	7.3371E-06	Bilgir (Turkey)
0.6884	0.6566	0.7185	0.7887	0.7545	0.8192	9.3339E-06	Wang F (Wuhan, China)

Supplementary Table 3:

Pooled AUCs for D-dimer calculated with omitting a single study at a time. The first line (in bold) shows the summary from all studies with no omissions.

Pooled AUC Europe/NA	Lower Limit Europe/NA	Upper Limit Europe/NA	Pooled AUC Asia	Lower Limit Asia	Upper Limit Asia	p-value	Study omitted
**0.7791**	**0.7431**	**0.8114**	**0.7856**	**0.6958**	**0.8544**	**0.86183**	**None**
0.7846	0.7438	0.8205	0.7856	0.6958	0.8544	0.97986	Soon (Cambridge)
0.7615	0.7182	0.8000	0.7856	0.6958	0.8544	0.53160	Soares (Spain)
0.7784	0.7315	0.8192	0.7856	0.6958	0.8544	0.85104	Kaufmann (Austria)
0.7911	0.7573	0.8213	0.7856	0.6958	0.8544	0.87794	van Dam (Netherlands)
0.7778	0.7435	0.8086	0.7856	0.6958	0.8544	0.83132	Carlino (Italy)
0.7768	0.7267	0.8200	0.7856	0.6958	0.8544	0.82051	Gomez (Spain)
0.7791	0.7431	0.8114	0.7961	0.6938	0.8707	0.67502	Asghar (Pakistan)
0.7791	0.7431	0.8114	0.8077	0.7299	0.8672	0.38689	Tahtasakal (Turkey)
0.7791	0.7431	0.8114	0.7893	0.6826	0.8671	0.80740	Ke (China)
0.7791	0.7431	0.8114	0.7754	0.6712	0.8538	0.92743	Zhang J (China)
0.7791	0.7431	0.8114	0.7782	0.6748	0.8557	0.98167	Liu Q (China)
0.7791	0.7431	0.8114	0.7989	0.7099	0.8657	0.58671	Cheng S (China)
0.7791	0.7431	0.8114	0.7646	0.6668	0.8406	0.70703	Cheng A (China)
0.7791	0.7431	0.8114	0.7778	0.6734	0.8560	0.97432	Zeng HL (China)
0.7791	0.7431	0.8114	0.7807	0.6837	0.8543	0.96784	Bilgir (Turkey)

Supplementary Table 4

Pooled AUCs for urea calculated with omitting a single study at a time. The first line (in bold) shows the summary from all studies with no omissions.

Pooled AUC Europe/NA	Lower Limit Europe/NA	Upper Limit Europe/NA	Pooled AUC Asia	Lower Limit Asia	Upper Limit Asia	p-value	Study omitted
**0.7891**	**0.7415**	**0.8299**	**0.8121**	**0.7659**	**0.8509**	**0.42437**	**None**
0.7934	0.7430	0.8361	0.8121	0.7659	0.8509	0.52584	Soon (Cambridge)
0.7850	0.7337	0.8287	0.8121	0.7659	0.8509	0.36282	Soares (Spain)
0.7852	0.7344	0.8286	0.8121	0.7659	0.8509	0.36515	Kaufmann (Austria)
0.7881	0.7362	0.8320	0.8121	0.7659	0.8509	0.42128	de Michieli (Italy)
0.7869	0.7353	0.8308	0.8121	0.7659	0.8509	0.39882	Peiro (Spain)
0.7875	0.7366	0.8308	0.8121	0.7659	0.8509	0.40640	Cipriani (Italy)
0.7969	0.7500	0.8370	0.8121	0.7659	0.8509	0.59410	Smilowitz (USA)
0.7862	0.7361	0.8290	0.8121	0.7659	0.8509	0.37902	Goudot (France)
0.7950	0.7462	0.8365	0.8121	0.7659	0.8509	0.55548	Macias-Munoz (Spain)
0.7969	0.7523	0.8352	0.8121	0.7659	0.8509	0.58517	Myrhe (Norway)
0.7895	0.7376	0.8335	0.8121	0.7659	0.8509	0.45003	Harmouch (USA)
0.7899	0.7379	0.8339	0.8121	0.7659	0.8509	0.45753	Manocha (USA)
0.7929	0.7427	0.8354	0.8121	0.7659	0.8509	0.51268	Herold (Germany)
0.7775	0.7364	0.8138	0.8121	0.7659	0.8509	0.20345	Aloisio (Italy)
0.7928	0.7425	0.8355	0.8121	0.7659	0.8509	0.51208	Petersen-Uribe (Germany)
0.7824	0.7318	0.8256	0.8121	0.7659	0.8509	0.31472	Salvatici (Italy)
0.7891	0.7415	0.8299	0.8184	0.7728	0.8566	0.30315	Asghar (Pakistan)
0.7891	0.7415	0.8299	0.8088	0.7588	0.8504	0.50928	Deng (China)
0.7891	0.7415	0.8299	0.8174	0.7696	0.8572	0.33105	Tahtasakal (Turkey)
0.7891	0.7415	0.8299	0.8115	0.7614	0.8532	0.45270	Liang (China)
0.7891	0.7415	0.8299	0.8088	0.7591	0.8502	0.50742	Chen L (China)
0.7891	0.7415	0.8299	0.8092	0.7605	0.8500	0.49423	Cao (China)
0.7891	0.7415	0.8299	0.8180	0.7707	0.8573	0.31939	Chen H (China)
0.7891	0.7415	0.8299	0.8136	0.7638	0.8548	0.41108	Wang Y (China)
0.7891	0.7415	0.8299	0.8119	0.7635	0.8523	0.43622	Ozyilmaz (Turkey)
0.7891	0.7415	0.8299	0.8164	0.7688	0.8560	0.34777	Zhang J (China)
0.7891	0.7415	0.8299	0.8072	0.7573	0.8489	0.54380	Luo Y (China)
0.7891	0.7415	0.8299	0.8208	0.7782	0.8566	0.25043	Ozdin (Turkey)
0.7891	0.7415	0.8299	0.8108	0.7605	0.8526	0.46780	Sun W (Wuhan, China)
0.7891	0.7415	0.8299	0.8029	0.7581	0.8412	0.62438	Shi S (Wuhan, China)
0.7891	0.7415	0.8299	0.8146	0.7651	0.8556	0.39030	Qin JJ (Wuhan, China)
0.7891	0.7415	0.8299	0.8014	0.7549	0.8410	0.66842	Zhu F (Shanghai, China)

Supplementary Table 5

Pooled AUCs for troponin calculated with omitting a single study at a time. The first line (in bold) shows the summary from all studies with no omissions.

Pooled AUC Europe/NA	Lower Limit Europe/NA	Upper Limit Europe/NA	Pooled AUC Asia	Lower Limit Asia	Upper Limit Asia	p-value	Study omitted
**0.69910**	**0.64283**	**0.74995**	**0.86064**	**0.80530**	**0.90216**	**8.76545E-06**	**None**
0.70017	0.63808	0.75570	0.86064	0.80530	0.90216	1.73829E-05	Soon (Cambridge)
0.69989	0.63872	0.75468	0.86064	0.80530	0.90216	1.5351E-05	Soares (Spain)
0.69206	0.63350	0.74503	0.86064	0.80530	0.90216	4.97628E-06	Maeda (USA)
0.69755	0.63714	0.75182	0.86064	0.80530	0.90216	1.1052E-05	Nagant (Belgium)
0.70750	0.65397	0.75584	0.86064	0.80530	0.90216	1.61647E-05	Pieri (Italy)
0.70545	0.64717	0.75771	0.86064	0.80530	0.90216	2.07354E-05	Kara (USA)
0.69668	0.63566	0.75147	0.86064	0.80530	0.90216	1.073E-05	Laguna-Goya (Spain)
0.70349	0.64457	0.75634	0.86064	0.80530	0.90216	1.79307E-05	Mueller (USA)
0.70895	0.65978	0.75367	0.86064	0.80530	0.90216	1.23311E-05	Donoso-Navarro (Spain)
0.70087	0.63878	0.75635	0.86064	0.80530	0.90216	1.86744E-05	Muinos (Spain)
0.69659	0.64033	0.74752	0.86064	0.80530	0.90216	6.40601E-06	Carlino (Italy)
0.68459	0.63518	0.73016	0.86064	0.80530	0.90216	7.25907E-07	Herold (Germany)
0.69130	0.63462	0.74276	0.86064	0.80530	0.90216	3.70937E-06	Quartuccio (Italy)
0.70026	0.63890	0.75519	0.86064	0.80530	0.90216	1.62707E-05	Myrhe (Norway)
0.69768	0.63823	0.75117	0.86064	0.80530	0.90216	1.01345E-05	Guirao (Spain)
0.70449	0.64556	0.75730	0.86064	0.80530	0.90216	1.99393E-05	Villa (Italy)
0.69800	0.63722	0.75255	0.86064	0.80530	0.90216	1.20541E-05	Ruscica (Italy)
0.69910	0.64283	0.74995	0.86179	0.79323	0.91019	7.37739E-05	Shi (China)
0.69910	0.64283	0.74995	0.86602	0.80686	0.90910	1.03533E-05	Ke (China)
0.69910	0.64283	0.74995	0.85088	0.79167	0.89548	4.6002E-05	Shang (China)
0.69910	0.64283	0.74995	0.87035	0.81733	0.90968	1.44626E-06	Zhang J (China)
0.69910	0.64283	0.74995	0.84044	0.79690	0.87609	5.4559E-06	Zhang (China)
0.69910	0.64283	0.74995	0.86650	0.80807	0.90914	8.39488E-06	Pan (China)
0.69910	0.64283	0.74995	0.86126	0.79939	0.90629	2.8198E-05	Han (China)
0.69910	0.64283	0.74995	0.86179	0.79323	0.91019	7.37739E-05	Shi (China)
0.69910	0.64283	0.74995	0.85909	0.80003	0.90283	1.99223E-05	Satis (Turkey)
0.69910	0.64283	0.74995	0.86546	0.80704	0.90821	9.28051E-06	Gao Y (China)
0.69910	0.64283	0.74995	0.86038	0.79911	0.90517	2.7228E-05	Saji (Japan)
0.69910	0.64283	0.74995	0.85836	0.79583	0.90404	4.15284E-05	Wang F (China)

Supplementary Table 6

Pooled AUCs for IL-6 calculated with omitting a single study at a time. The first line (in bold) shows the summary from all studies with no omissions.

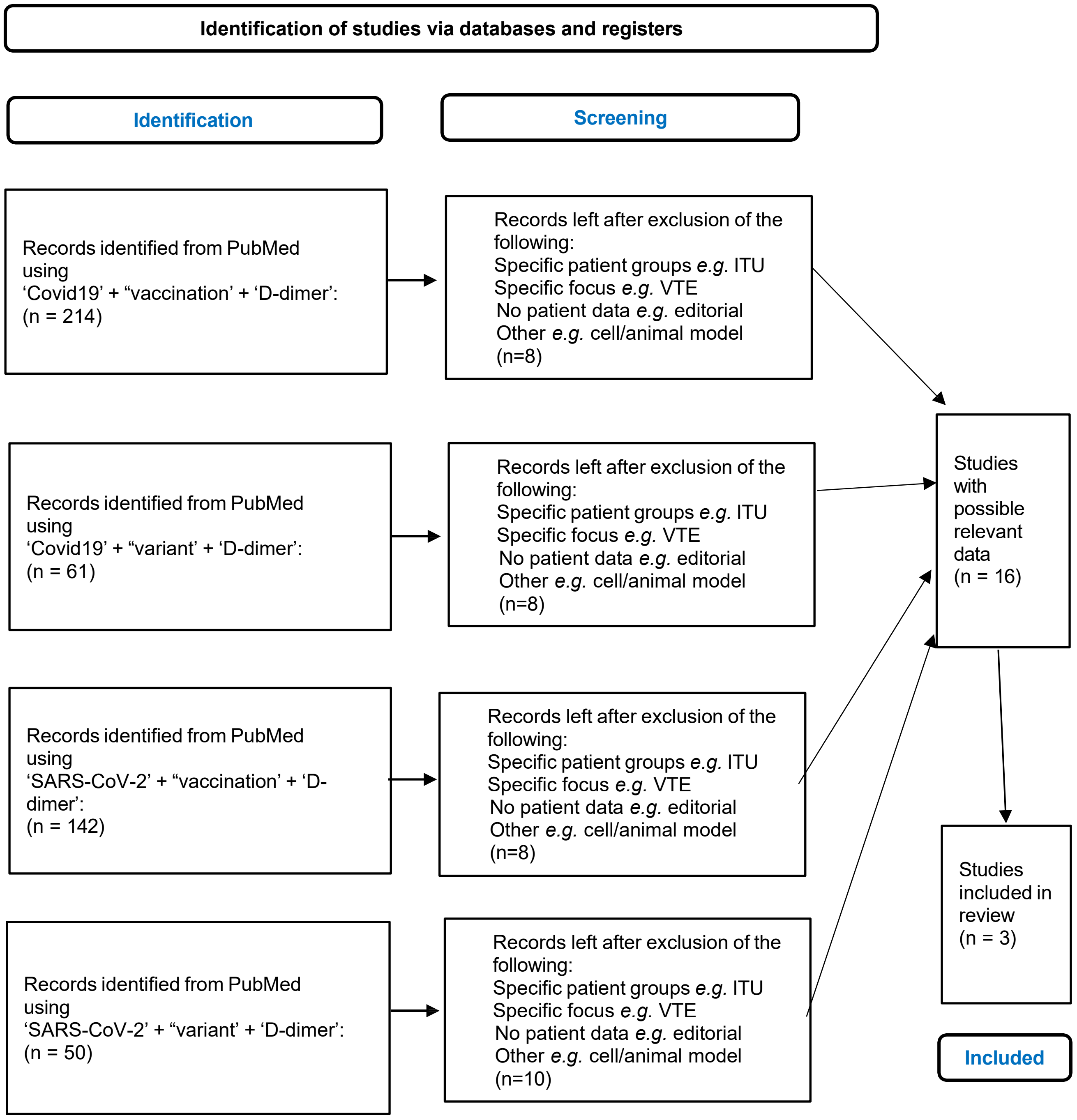

Supplementary Figure 1

Flow diagram for identification and selection of studies regarding utility of D-dimer as a biomarker in vaccinated and/or variant cohorts.

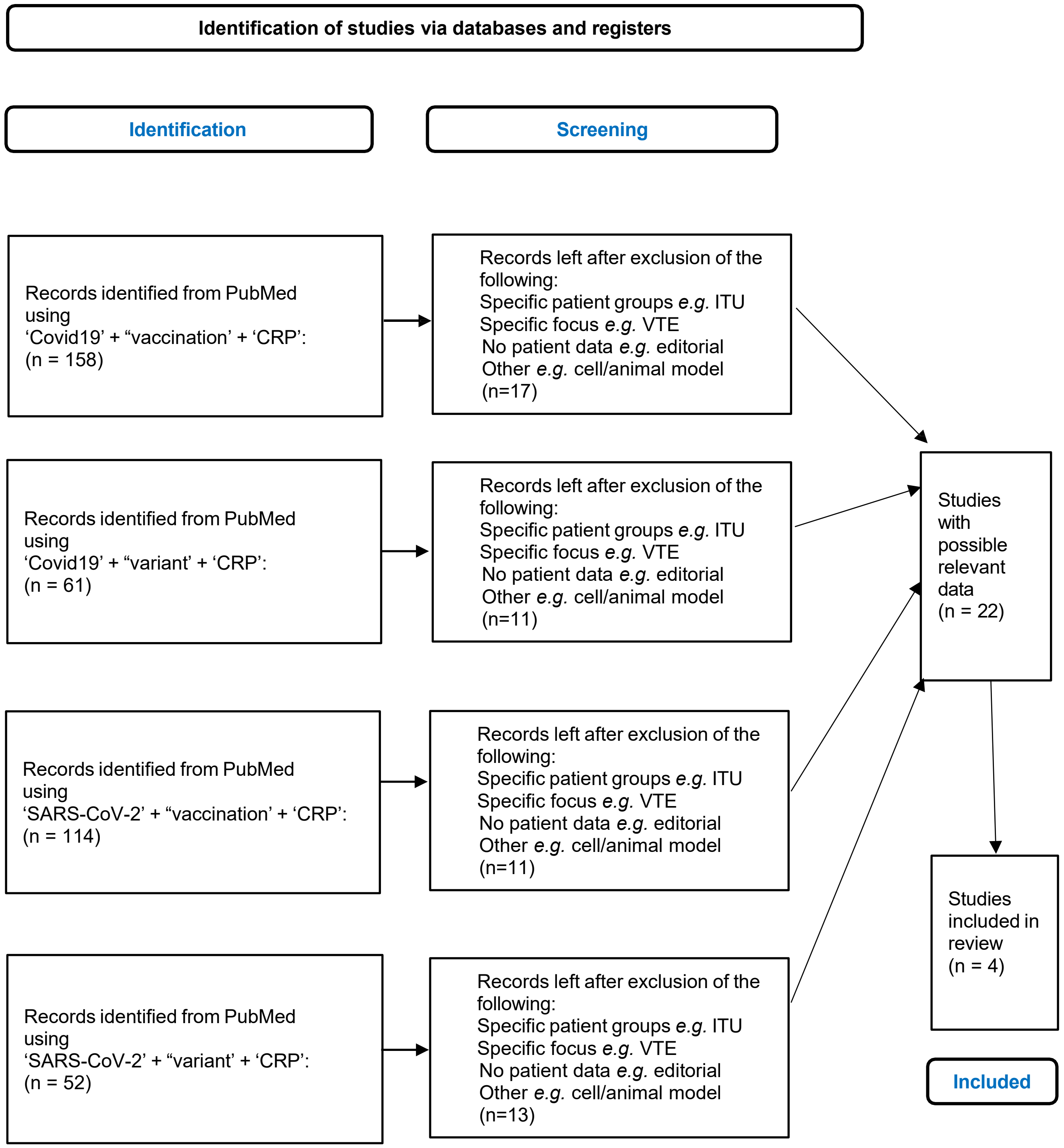

Supplementary Figure 2

Flow diagram for identification and selection of studies regarding utility of CRP as a biomarker in vaccinated and/or variant cohorts.

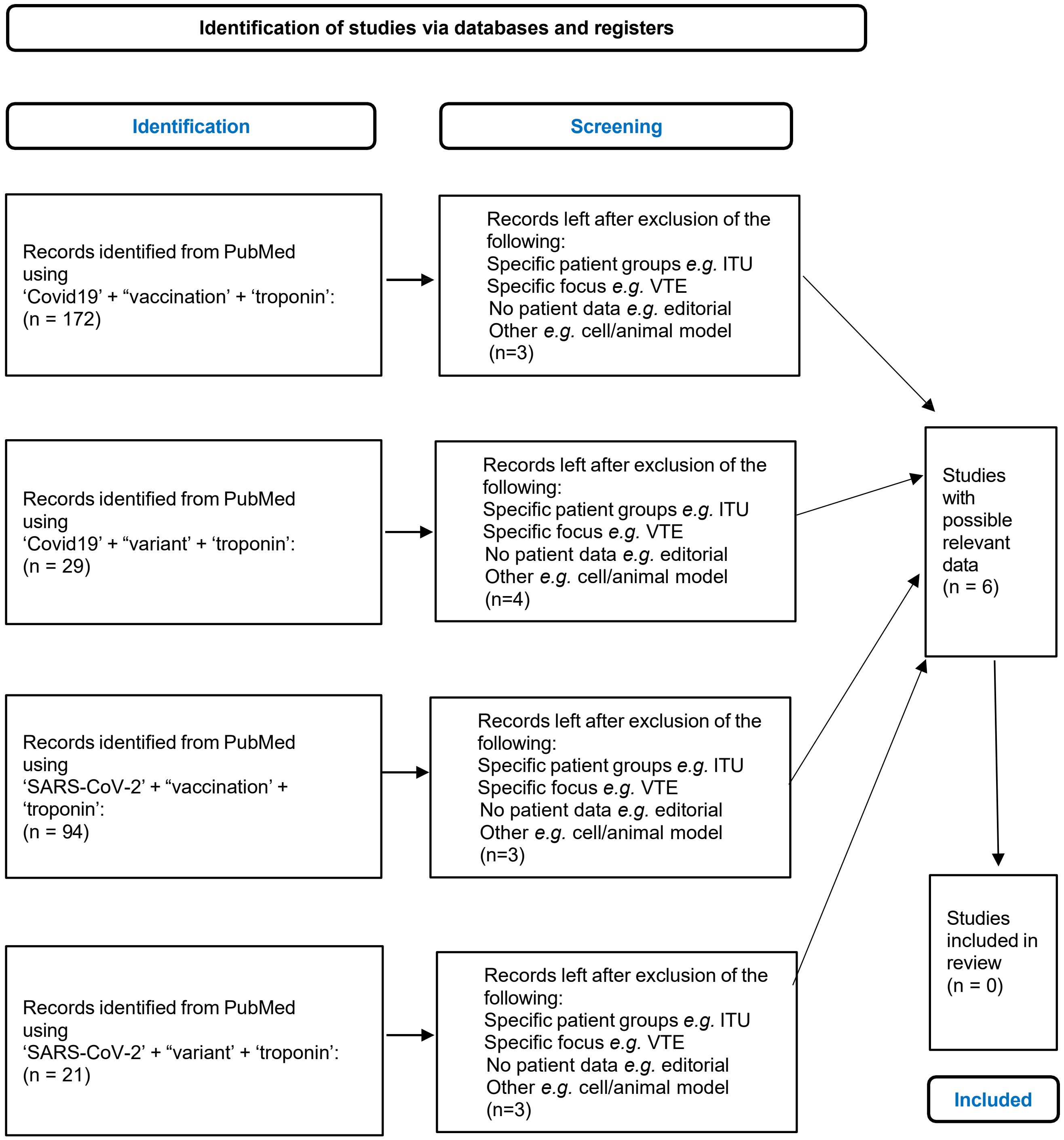

Supplementary Figure 3

Flow diagram for identification and selection of studies regarding utility of troponin as a biomarker in vaccinated and/or variant cohorts.

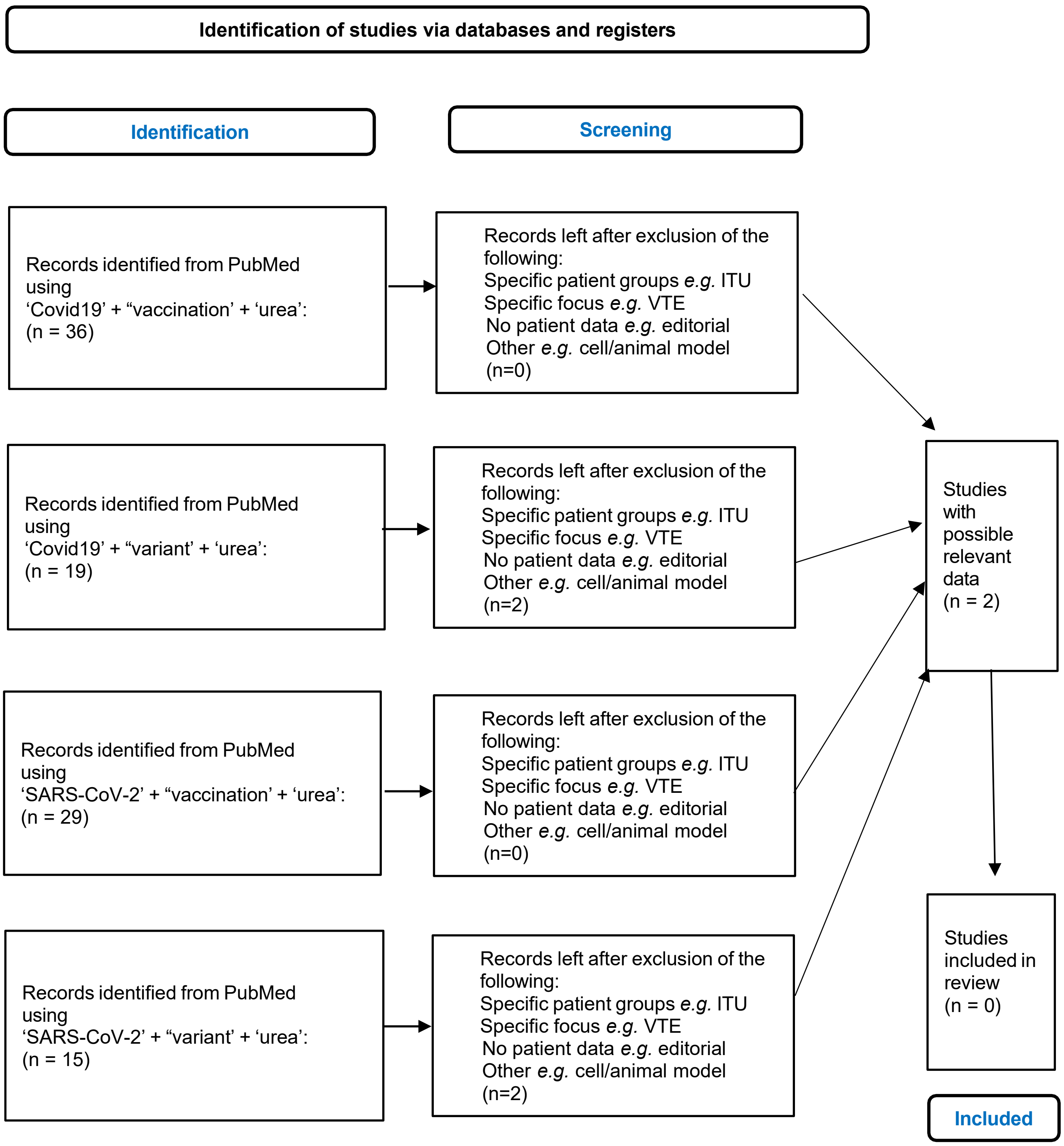

Supplementary Figure 4

Flow diagram for identification and selection of studies regarding utility of urea as a biomarker in vaccinated and/or variant cohorts.

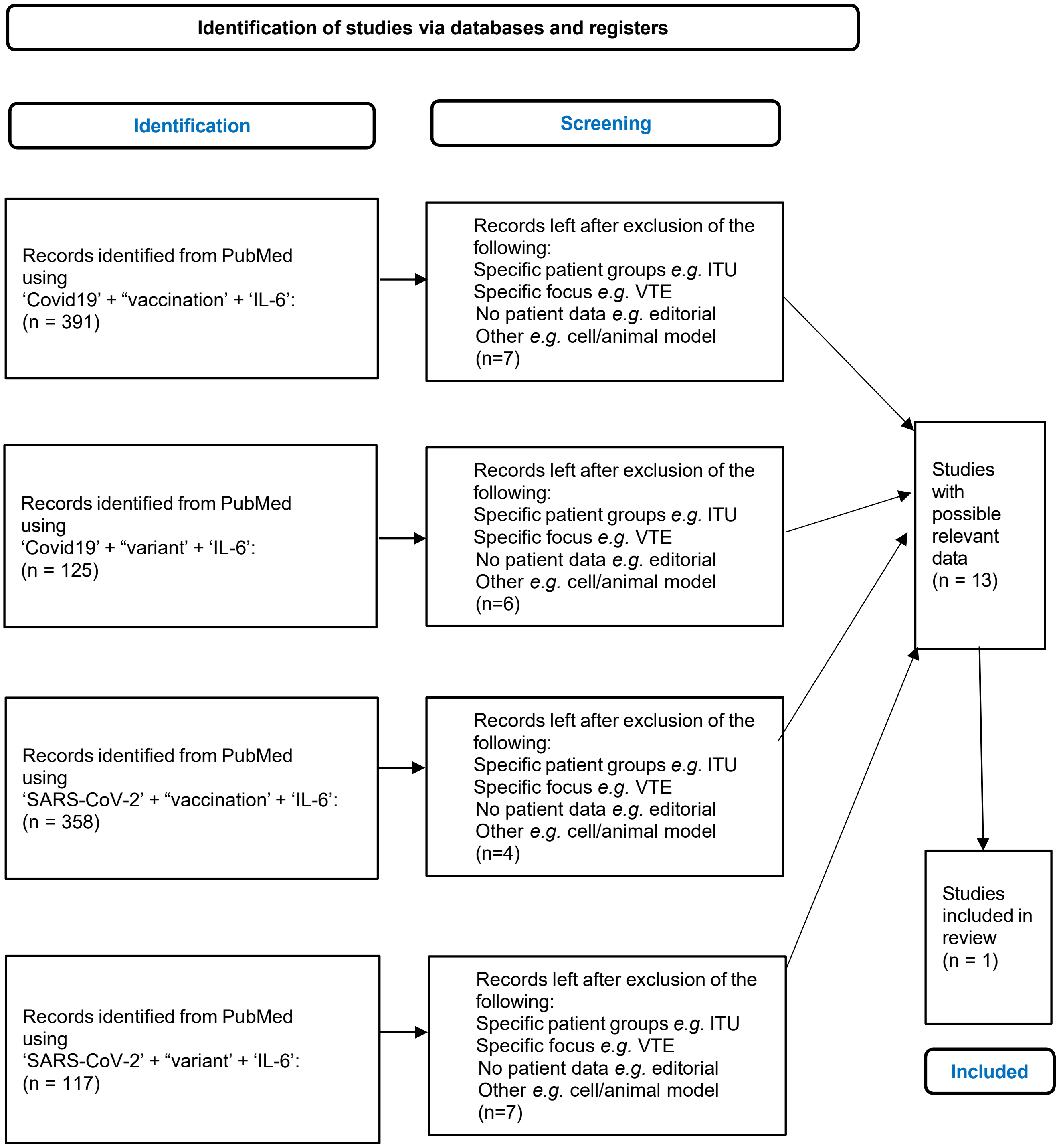

Supplementary Figure 5

Flow diagram for identification and selection of studies regarding utility of IL-6 as a biomarker in vaccinated and/or variant cohorts.

References

1. Aloisio, E., et al. (2020). “A Comprehensive Appraisal of Laboratory Biochemistry Tests as Major Predictors of COVID-19 Severity.” Arch Pathol Lab Med 144(12): 1457-1464.
2. Antunez Muiños, P. J., et al. (2021). “The COVID-19 lab score: an accurate dynamic tool to predict in-hospital outcomes in COVID-19 patients.” Sci Rep 11(1): 9361.
3. Asghar, M. S., et al. (2020). “Poor Prognostic Biochemical Markers Predicting Fatalities Caused by COVID-19: A Retrospective Observational Study From a Developing Country.” Cureus 12(8): e9575.
4. Bastug, A., et al. (2020). “Clinical and laboratory features of COVID-19: Predictors of severe prognosis.” Int Immunopharmacol 88: 106950.
5. Cao, J., et al. (2020). “Myocardial injury and COVID-19: Serum hs-cTnI level in risk stratification and the prediction of 30-day fatality in COVID-19 patients with no prior cardiovascular disease.” Theranostics 10(21): 9663-9673.
6. Carlino, M. V., et al. (2020). “Predictors of Intensive Care Unit admission in patients with coronavirus disease 2019 (COVID-19).” Monaldi Arch Chest Dis 90(3).
7. Chen, F. F., et al. (2020). “The characteristics and outcomes of 681 severe cases with COVID-19 in China.” J Crit Care 60: 32-37.
8. Chen, H., et al. (2021). “Cardiac Troponin I association with critical illness and death risk in 726 seriously ill COVID-19 patients: A retrospective cohort study.” Int J Med Sci 18(6): 1474-1483.
9. Chen, L., et al. (2021). “Association of coagulation dysfunction with cardiac injury among hospitalized patients with COVID-19.” Sci Rep 11(1): 4432.
10. Chen, L., et al. (2020). “Risk factors for death in 1859 subjects with COVID-19.” Leukemia 34(8): 2173-2183.
11. Chen, X., et al. (2020). “A Retrospective Analysis of the Coagulation Dysfunction in COVID-19 Patients.” Clin Appl Thromb Hemost 26: 1076029620964868.
12. Cheng, A., et al. (2020). “Diagnostic performance of initial blood urea nitrogen combined with D-dimer levels for predicting in-hospital mortality in COVID-19 patients.” Int J Antimicrob Agents 56(3): 106110.
13. Cheng, B., et al. (2020). “Predictors of progression from moderate to severe coronavirus disease 2019: a retrospective cohort.” Clin Microbiol Infect 26(10): 1400-1405.
14. Cheng, S., et al. (2020). “Risk factors for the critical illness in SARS-CoV-2 infection: a multicenter retrospective cohort study.” Respir Res 21(1): 277.

15. Chocron, R., et al. (2021). "D-dimer at hospital admission for COVID-19 are associated with in-hospital mortality, independent of venous thromboembolism: Insights from a French multicenter cohort study." Arch Cardiovasc Dis.

16. Cipriani, A., et al. (2021). "Cardiac injury and mortality in patients with Coronavirus disease 2019 (COVID-19): insights from a mediation analysis." Intern Emerg Med 16(2): 419-427.

17. Creel-Bulos, C., et al. (2020). "Trends and diagnostic value of D-dimer levels in patients hospitalized with coronavirus disease 2019." Medicine (Baltimore) 99(46): e23186.

18. De Michieli, L., et al. (2021). "Using high sensitivity cardiac troponin values in patients with SARS-CoV-2 infection (COVID-19): The Padova experience." Clin Biochem 90: 8-14.

19. Deng, P., et al. (2020). "The diagnostic and prognostic role of myocardial injury biomarkers in hospitalized patients with COVID-19." Clin Chim Acta 510: 186-190.

20. Donoso-Navarro, E., et al. (2021). "IL-6 and Other Biomarkers associated with Poor Prognosis in a Cohort of Hospitalized Patients with COVID-19 in Madrid." Biomark Insights 16: 11772719211013363.

21. Duan, J., et al. (2020). "Correlation between the variables collected at admission and progression to severe cases during hospitalization among patients with COVID-19 in Chongqing." J Med Virol 92(11): 2616-2622.

22. El-Solh, A. A., et al. (2020). "Comparison of in-hospital mortality risk prediction models from COVID-19." PLoS One 15(12): e0244629.

23. El-Solh, A. A., et al. (2021). "Clinical Course and Outcome of COVID-19 Acute Respiratory Distress Syndrome: Data From a National Repository." J Intensive Care Med 36(6): 664-672.

24. Fu, J., et al. (2020). "The clinical implication of dynamic neutrophil to lymphocyte ratio and D-dimer in COVID-19: A retrospective study in Suzhou China." Thromb Res 192: 3-8.

25. Gao, Y., et al. (2020). "Diagnostic utility of clinical laboratory data determinations for patients with the severe COVID-19." J Med Virol 92(7): 791-796.

26. Goudot, G., et al. (2020). "Predictive Factor for COVID-19 Worsening: Insights for High-Sensitivity Troponin and D-Dimer and Correlation With Right Ventricular Afterload." Front Med (Lausanne) 7: 586307.

27. Gu, Y., et al. (2021). "PaO(2)/FiO(2) and IL-6 are risk factors of mortality for intensive care COVID-19 patients." Sci Rep 11(1): 7334.

28. Gupta, R. K., et al. (2021). "Development and validation of the ISARIC 4C Deterioration model for adults hospitalised with COVID-19: a prospective cohort study." Lancet Respir Med 9(4): 349-359.

29. Han, H., et al. (2020). "Profiling serum cytokines in COVID-19 patients reveals IL-6 and IL-10 are

disease severity predictors." Emerg Microbes Infect 9(1): 1123-1130.

30. Harmouch, F., et al. (2021). "Is it all in the heart? Myocardial injury as major predictor of mortality among hospitalized COVID-19 patients." J Med Virol 93(2): 973-982.

31. He, X., et al. (2021). "The poor prognosis and influencing factors of high D-dimer levels for COVID-19 patients." Sci Rep 11(1): 1830.

32. Herold, T., et al. (2020). "Elevated levels of IL-6 and CRP predict the need for mechanical ventilation in COVID-19." J Allergy Clin Immunol 146(1): 128-136.e124.

33. Huang, Y., et al. (2020). "A cohort study of 676 patients indicates D-dimer is a critical risk factor for the mortality of COVID-19." PLoS One 15(11): e0242045.

34. Huff, H. V. and A. Singh (2020). "Asymptomatic Transmission During the Coronavirus Disease 2019 Pandemic and Implications for Public Health Strategies." Clin Infect Dis 71(10): 2752-2756.

35. Infante, M., et al. (2021). "Low Vitamin D Status at Admission as a Risk Factor for Poor Survival in Hospitalized Patients With COVID-19: An Italian Retrospective Study." J Am Coll Nutr: 1-16.

36. Jin, X., et al. (2020). "The values of coagulation function in COVID-19 patients." PLoS One 15(10): e0241329.

37. Jøntvedt Jørgensen, M., et al. (2020). "Increased interleukin-6 and macrophage chemoattractant protein-1 are associated with respiratory failure in COVID-19." Sci Rep 10(1): 21697.

38. Kaufmann, C. C., et al. (2021). "Mid-regional pro-atrial natriuretic peptide independently predicts short-term mortality in COVID-19." Eur J Clin Invest 51(5): e13531.

39. Ke, C., et al. (2020). "Clinical characteristics of confirmed and clinically diagnosed patients with 2019 novel coronavirus pneumonia: a single-center, retrospective, case-control study." Med Clin (Engl Ed) 155(8): 327-334.

40. Keski, H. (2021). "Hematological and Inflammatory Parameters to Predict the Prognosis in COVID-19." Indian J Hematol Blood Transfus: 1-9.

41. Küçükceran, K., et al. (2021). "Predictive value of D-dimer/albumin ratio and fibrinogen/albumin ratio for in-hospital mortality in patients with COVID-19." Int J Clin Pract: e14263.

42. Laguna-Goya, R., et al. (2020). "IL-6-based mortality risk model for hospitalized patients with COVID-19." J Allergy Clin Immunol 146(4): 799-807.e799.

43. Liang, M., et al. (2020). "Novel risk scoring system for predicting acute respiratory distress syndrome among hospitalized patients with coronavirus disease 2019 in Wuhan, China." BMC Infect Dis 20(1): 960.

44. Liu, F., et al. (2020). "Prognostic value of interleukin-6, C-reactive protein, and procalcitonin in patients

with COVID-19." J Clin Virol 127: 104370.

45. Liu, Q., et al. (2020). "Laboratory findings and a combined multifactorial approach to predict death in critically ill patients with COVID-19: a retrospective study." Epidemiol Infect 148: e129.

46. Liu, S. L., et al. (2020). "Expressions of SAA, CRP, and FERR in different severities of COVID-19." Eur Rev Med Pharmacol Sci 24(21): 11386-11394.

47. Long, H., et al. (2020). "D-Dimer and Prothrombin Time Are the Significant Indicators of Severe COVID-19 and Poor Prognosis." Biomed Res Int 2020: 6159720.

48. Luo, H. C., et al. (2021). "Characteristics of coagulation alteration in patients with COVID-19." Ann Hematol 100(1): 45-52.

49. Luo, X., et al. (2020). "Prognostic Value of C-Reactive Protein in Patients With Coronavirus 2019." Clin Infect Dis 71(16): 2174-2179.

50. Luo, Y., et al. (2020). "Prealbumin as a Predictor of Prognosis in Patients With Coronavirus Disease 2019." Front Med (Lausanne) 7: 374.

51. Macias-Muñoz, L., et al. (2021). "Value of clinical laboratory test for early prediction of mortality in patients with COVID-19: the BGM score." J Circ Biomark 10: 1-8.

52. Maeda, T., et al. (2021). "The association of interleukin-6 value, interleukin inhibitors, and outcomes of patients with COVID-19 in New York City." J Med Virol 93(1): 463-471.

53. Manocha, K. K., et al. (2021). "Troponin and Other Biomarker Levels and Outcomes Among Patients Hospitalized With COVID-19: Derivation and Validation of the HA(2)T(2) COVID-19 Mortality Risk Score."

54. Montrucchio, G., et al. (2021). "Effectiveness of mid-regional pro-adrenomedullin (MR-proADM) as prognostic marker in COVID-19 critically ill patients: An observational prospective study." PLoS One 16(2): e0246771.

55. Mueller, A. A., et al. (2020). "Inflammatory Biomarker Trends Predict Respiratory Decline in COVID-19 Patients." Cell Rep Med 1(8): 100144.

56. Muhammad, R., et al. (2021). "Clinical Characteristics and Predictors of Mortality in Minority Patients Hospitalized with COVID-19 Infection." J Racial Ethn Health Disparities: 1-11.

57. Myhre, P. L., et al. (2020). "Growth Differentiation Factor 15 Provides Prognostic Information Superior to Established Cardiovascular and Inflammatory Biomarkers in Unselected Patients Hospitalized With COVID-19." Circulation 142(22): 2128-2137.

58. Nagant, C., et al. (2020). "A score combining early detection of cytokines accurately predicts COVID-19 severity and intensive care unit transfer." Int J Infect Dis 101: 342-345.

59. Nascimento, J. H. P., et al. (2021). "COVID-19 and Myocardial Injury in a Brazilian ICU: High Incidence and Higher Risk of In-Hospital Mortality." Arq Bras Cardiol 116(2): 275-282.

60. Naymagon, L., et al. (2020). "Admission D-dimer levels, D-dimer trends, and outcomes in COVID-19." Thromb Res 196: 99-105.

61. Otoshi, R., et al. (2021). "Clinical characteristics of Japanese patients with moderate to severe COVID-19." J Infect Chemother 27(6): 895-901.

62. Özyılmaz, S., et al. (2020). "Assessment of the Relationship between Mortality and Troponin I Levels in Hospitalized Patients with the Novel Coronavirus (COVID-19)." Medicina (Kaunas) 56(12).

63. Pan, M., et al. (2021). "Laboratory predictors of severe Coronavirus Disease 2019 and lung function in followed-up." Clin Respir J.

64. Pascual Gómez, N. F., et al. (2020). "[Potential biomarkers predictors of mortality in COVID-19 patients in the Emergency Department]." Rev Esp Quimioter 33(4): 267-273.

65. Patel, K. P., et al. (2020). "Transmission of SARS-CoV-2: an update of current literature." Eur J Clin Microbiol Infect Dis 39(11): 2005-2011.

66. Peiró Ó, M., et al. (2021). "Biomarkers and short-term prognosis in COVID-19." Biomarkers 26(2): 119-126.

67. Pieri, M., et al. (2021). "Serum Amyloid A Protein as a useful biomarker to predict COVID-19 patients severity and prognosis." Int Immunopharmacol 95: 107512.

68. Pouw, N., et al. (2021). "Clinical characteristics and outcomes of 952 hospitalized COVID-19 patients in The Netherlands: A retrospective cohort study." PLoS One 16(3): e0248713.

69. Qin, Z. J., et al. (2020). "Impaired immune and coagulation systems may be early risk factors for COVID-19 patients: A retrospective study of 118 inpatients from Wuhan, China." Medicine (Baltimore) 99(35): e21700.

70. Quartuccio, L., et al. (2021). "Interleukin 6, soluble interleukin 2 receptor alpha (CD25), monocyte colony-stimulating factor, and hepatocyte growth factor linked with systemic hyperinflammation, innate immunity hyperactivation, and organ damage in COVID-19 pneumonia." Cytokine 140: 155438.

71. Rubio-Rivas, M., et al. (2021). "Risk Categories in COVID-19 Based on Degrees of Inflammation: Data on More Than 17,000 Patients from the Spanish SEMI-COVID-19 Registry." J Clin Med 10(10).

72. Salinas, M., et al. (2020). "Laboratory parameters in patients with COVID-19 on first emergency admission is different in non-survivors: albumin and lactate dehydrogenase as risk factors." J Clin Pathol.

73. Santa Cruz, A., et al. (2021). "Interleukin-6 Is a Biomarker for the Development of Fatal Severe Acute

Respiratory Syndrome Coronavirus 2 Pneumonia." Front Immunol 12: 613422.
74. Satış, H., et al. (2021). "Prognostic value of interleukin-18 and its association with other inflammatory markers and disease severity in COVID-19." Cytokine 137: 155302.
75. Selcuk, M., et al. (2021). "Comparison of D-dimer Level Measured on the Third Day of Hospitalization with Admission D-dimer Level in Predicting In-hospital Mortality in COVID-19 Patients." Medeni Med J 36(1): 1-6.
76. Shang, M., et al. (2021). "Early Warning Factors of Death in COVID-19 Patients." Curr Med Sci 41(1): 69-76.
77. Shang, W., et al. (2020). "The value of clinical parameters in predicting the severity of COVID-19." J Med Virol 92(10): 2188-2192.
78. Sharif, F., et al. (2021). "Early hematological indicators of severe COVID-19 disease in hospitalized patients: Data from a South Asian population." Int J Lab Hematol.
79. Shi, S., et al. (2021). "Clinical and laboratory characteristics of severe and non-severe patients with COVID-19: A retrospective cohort study in China." J Clin Lab Anal 35(1): e23692.
80. Shi, X., et al. (2020). "[Value of interleukin-6 and CD4(+) T-lymphocytopenia in assessing the severity and prognosis of coronavirus disease 2019]." Zhonghua Wei Zhong Bing Ji Jiu Yi Xue 32(10): 1165-1170.
81. Smilowitz, N. R., et al. (2021). "C-reactive protein and clinical outcomes in patients with COVID-19." Eur Heart J 42(23): 2270-2279.
82. Soni, M., et al. (2020). "D-dimer level is a useful predictor for mortality in patients with COVID-19: Analysis of 483 cases." Diabetes Metab Syndr 14(6): 2245-2249.
83. Tahtasakal, C. A., et al. (2021). "Could we predict the prognosis of the COVID-19 disease?" J Med Virol 93(4): 2420-2430.
84. Tian, W., et al. (2020). "Predictors of mortality in hospitalized COVID-19 patients: A systematic review and meta-analysis." J Med Virol 92(10): 1875-1883.
85. van Dam, P., et al. (2021). "Performance of prediction models for short-term outcome in COVID-19 patients in the emergency department: a retrospective study." Ann Med 53(1): 402-409.
86. Wang, D., et al. (2020). "Correlation analysis between disease severity and clinical and biochemical characteristics of 143 cases of COVID-19 in Wuhan, China: a descriptive study." BMC Infect Dis 20(1): 519.
87. Wang, M., et al. (2020). "Differences of inflammatory and non-inflammatory indicators in Coronavirus disease-19 (COVID-19) with different severity." Infect Genet Evol 85: 104511.
88. Wang, Y., et al. (2021). "The peak levels of highly sensitive troponin I predicts in-hospital mortality in

COVID-19 patients with cardiac injury: a retrospective study." Eur Heart J Acute Cardiovasc Care 10(1): 6-15.

89. Wang, Y., et al. (2020). "Cardiac Injury and Clinical Course of Patients With Coronavirus Disease 2019." Front Cardiovasc Med 7: 147.

90. Wang, Z. and Z. Wang (2021). "Identification of risk factors for in-hospital death of COVID – 19 pneumonia — lessions from the early outbreak." BMC Infect Dis 21(1): 113.

91. Xu, F., et al. (2021). "Prediction of Disease Progression of COVID-19 Based upon Machine Learning." Int J Gen Med 14: 1589-1598.

92. Yang, A. P., et al. (2020). "Infection with SARS-CoV-2 causes abnormal laboratory results of multiple organs in patients." Aging (Albany NY) 12(11): 10059-10069.

93. Yao, Y., et al. (2020). "D-dimer as a biomarker for disease severity and mortality in COVID-19 patients: a case control study." J Intensive Care 8: 49.

94. Ye, W., et al. (2020). "Dynamic changes of D-dimer and neutrophil-lymphocyte count ratio as prognostic biomarkers in COVID-19." Respir Res 21(1): 169.

95. Ye, Y., et al. (2021). "Prediction and follow-up of risk factors for severe SARS-CoV-2 pneumonia and application of CT visual scoring." Technol Health Care 29(S1): 153-164.

96. Yu, J., et al. (2020). "Prognostic Value of a Clinical Biochemistry-Based Nomogram for Coronavirus Disease 2019." Front Med (Lausanne) 7: 597791.

97. Yue, T., et al. (2021). "Combined clinical and imaging features better predict the critical outcomes of patients with SARS-COV-2." Medicine (Baltimore) 100(12): e25083.

98. Zayat, R., et al. (2020). "Role of extracorporeal membrane oxygenation in critically Ill COVID-19 patients and predictors of mortality." Artif Organs.

99. Zhang, J., et al. (2020). "Serum interleukin-6 is an indicator for severity in 901 patients with SARS-CoV-2 infection: a cohort study." J Transl Med 18(1): 406.

100. Zhang, J., et al. (2021). "Risk Factors for Mortality of COVID-19 Patient Based on Clinical Course: A Single Center Retrospective Case-Control Study." Front Immunol 12: 581469.

101. Zhang, L., et al. (2020). "D-dimer levels on admission to predict in-hospital mortality in patients with Covid-19." J Thromb Haemost 18(6): 1324-1329.

102. Zhao, Y., et al. (2021). "Peripheral blood inflammatory markers in predicting prognosis in patients with COVID-19. Some differences with influenza A." J Clin Lab Anal 35(1): e23657.

103. Zhu, Y., et al. (2020). "Evaluation of organ function in patients with severe COVID-19 infections." Med

Clin (Engl Ed) 155(5): 191-196

Acknowledgements

Many thanks to the following scientists and doctors who have interrogated their data further at our request.

Dr Jose Miguel Urra **Ardanaz**

Prof. Jeffrey **Berger**

Dr Francisco **Bernabeu Andreu**

Dr Alberto **Cipriani**

Dr Christina **Creel-Bulos**

Dr Tobias **Herold**

Dr Areeba **Kara** and Dr Warren **Gavin**

Dr Edy **Kim**

Dr Christoph **Kaufmann**

Dr Manuel **Morales**

Dr Peder Langeland **Myrhe**

Dr Carole **Nagant**

Dr Natalia **Pascual Gomez**

Mr Niels **Pouw** and Dr Jacobien **Hoogerwerf**

Dr Luca **Quartuccio**

Dr Morayma **Reyes Gil**

Dr Manuel **Rubio-Rivas**

Dr Maria **Salinas**

Dr Fatima **Sharif**

Dr Tao **Shen**

Dr Paul **van Dam**

Dr Erica **Villa**

Thank you!

We thank all patients and families affected by this pandemic who have contributed to the data needed for this work. We thank all healthcare, science, social care, and essential workers that have soldiered on, regardless of personal risk.
